# An intelligent LinkNet-34 model with EfficientNetB7 encoder for semantic segmentation of brain tumor

**DOI:** 10.1038/s41598-024-51472-2

**Published:** 2024-01-16

**Authors:** Adel Sulaiman, Vatsala Anand, Sheifali Gupta, Mana Saleh Al Reshan, Hani Alshahrani, Asadullah Shaikh, M. A. Elmagzoub

**Affiliations:** 1 Department of Computer Science, College of Computer Science and Information Systems, Najran University, 61441 Najran, Saudi Arabia; 2 Chitkara University Institute of Engineering and Technology, Chitkara University, Rajpura, Punjab 140401 India; 3 Department of Information Systems, College of Computer Science and Information Systems, Najran University, 61441 Najran, Saudi Arabia; 4 Department of Network and Communication Engineering, College of Computer Science and Information Systems, Najran University, 61441 Najran, Saudi Arabia

**Keywords:** Cancer, Health care

## Abstract

A brain tumor is an unnatural expansion of brain cells that can’t be stopped, making it one of the deadliest diseases of the nervous system. The brain tumor segmentation for its earlier diagnosis is a difficult task in the field of medical image analysis. Earlier, segmenting brain tumors was done manually by radiologists but that requires a lot of time and effort. Inspite of this, in the manual segmentation there was possibility of making mistakes due to human intervention. It has been proved that deep learning models can outperform human experts for the diagnosis of brain tumor in MRI images. These algorithms employ a huge number of MRI scans to learn the difficult patterns of brain tumors to segment them automatically and accurately. Here, an encoder-decoder based architecture with deep convolutional neural network is proposed for semantic segmentation of brain tumor in MRI images. The proposed method focuses on the image downsampling in the encoder part. For this, an intelligent LinkNet-34 model with EfficientNetB7 encoder based semantic segmentation model is proposed. The performance of LinkNet-34 model is compared with other three models namely FPN, U-Net, and PSPNet. Further, the performance of EfficientNetB7 used as encoder in LinkNet-34 model has been compared with three encoders namely ResNet34, MobileNet_V2, and ResNet50. After that, the proposed model is optimized using three different optimizers such as RMSProp, Adamax and Adam. The LinkNet-34 model has outperformed with EfficientNetB7 encoder using Adamax optimizer with the value of jaccard index as 0.89 and dice coefficient as 0.915.

## Introduction

A brain tumor is an abnormal growth of cells within the brain. Brain tumors pose significant health challenges due to their potential to disrupt normal brain functions and cause various neurological symptoms. Primary and secondary brain tumors are the most common subtypes. Secondary tumors can arise anywhere in the body and spread to the brain, whereas primary tumors always begin in the brain or the membranes that surround and protect the brain^1,2^. Depending on its size, location, and nature, the symptoms of a brain tumor may seem different. Complications that a brain tumor include are headaches, sickness and throwing up, issues with sight, and mental deterioration^3,4^.

Brain tumor segmentation is the process of accurately delineating and characterizing the tumor region in MRI scans of the brain. Segmentation is an essential step in medical image processing for accurately detecting brain tumors at an early stage. The difficulty arises from the intricate arrangement of brain design, the wide range of tumor types, and the common occurrence of distortions in MRI scans^5^. The main objective is to separate the tumor from the healthy brain tissue by dividing a complete brain MRI scan into segments that are smaller. Precise segmentation is crucial for accurately diagnosing brain tumors, devising treatment plans, and monitoring their progression. The segmentation procedure might be challenging due to the wide range of tumor forms, sizes, and locations, in addition to the existence of noise and anomalies in MRI images.

Both conventional image processing techniques and newer machine learning and deep learning methods have been created to address this task. During manual process of segmentation a radiologist employs a manual approach to precisely identify and outline a tumor in the MRI scan. This approach is labor-intensive, time-consuming, and prone to potential conflicts among observers. To autonomously segment the tumor area, automatic segmentation techniques used machine learning algorithms^6–8^. There are a number of benefits to using deep learning algorithms in brain tumor segmentation. It speeds up the diagnostic procedure, lowers the need for human intervention, and produces reliable, repeatable results every time^9,10^. As an added bonus, these robotic systems can help doctors spot even the most imperceptible tumor borders and sub-regions, which in turn allows for more accurate treatment planning and tracking.

The rest of the paper proceeds as follows: Section "Literature review" provides a summary of the relevant literature, Section "Proposed methodology" describes the proposed methodology, followed by results and discussion in Section "Results and discussion" and Section "Conclusions" draws a conclusion.

## Literature review

The existing literature approaches are studied here. Ramasamy et al.^11^ presented a deep learning model for semantic segmentation using a modified LinkNet-34 model. They used a multi-modal brain MRI dataset and obtained the value of accuracy as 99.2%. Cui et al.^12^ presented a cascaded deep CNN model using brain tumor segmentation datasets. They obtained the dice coefficient value of 0.89 with the combination of high and low-grade glioma. Corso et al.^13^ presented a hybrid method with a generative model. They worked on 20 cases of brain tumors and obtained the value of Jaccard as 0.62–0.69. Hamamci et al.^14^ proposed cellular automata model with a probability structure. They had worked on synthetic data from Harvard and had achieved the value of the dice coefficient as 0.72. Mehmood et al.^15^ presented a lesion localization and segmentation model using brain web data. They obtained a value of accuracy between 83 and 95%. Using the MICCAI brain dataset, authors^16–19^ designed various techniques and achieved the values of dice scores as 0.86, 0.88, 0.88, and 0.83 respectively.

Urban et al.^20^ proposed a 3D CNN model using the MICCAI brain dataset and attained the value of a dice coefficient of 0.87. Zikic et al.^21^ described a sliding kernel 3D network and worked on the MICCAI dataset. They had achieved the value of a dice coefficient as 0.84. Davy et al.^22^ presented global and local CNN to obtain the dice value of 0.85. Dvorak et al.^23^ had shown structured prediction using CNN. They obtained the value of the complete dice score as 0.83. Pereira et al.^24^ presented a CNN with small 3 * 3 kernels using the Brats dataset. They obtained the value of the complete dice score as 0.88. Havaei et al.^25^ presented the cascade neural network architecture using Brats dataset in 2019. They had achieved the value of a dice coefficient as 0.88. Lyksborg et al.^26^ used the 2014 Brats dataset to obtain the value of the complete dice score as 0.88. Kamnitsas et al.^27^ proposed the 2015-Brats dataset using 3D dense CNN to obtain the value of the complete dice score as 0.85. Zhu et al.^28^ had presented fusion of deep semantics and edge information in multimodal MRI by using Swin transformer to extract semantic features. They had worked using BraTS dataset with 989 MRI images of brain tumor. They had obtained the average value of dice coefficient as 86.72. Anusooya et al.^29^ had designed wavelet based attention network using channel attention and spatial attention modules. They had worked using 1251 patient’s data and had obtained the value of accuracy as 98%. Khan et al.^30^ had proposed a 3D deep learning based model with the help of lightweight feature extraction modules for brain tumor segmentation. They had worked using BraTs-2020 dataset and had achieved the value of dice-score as 0.867.

The major contributions of the study are as follows:

- An intelligent LinkNet-34 model with EfficientNetB7 encoder based semantic segmentation model is proposed for the segmentation of tumor from brain MRI images.
- The performance of LinkNet-34 model is compared with other three semantic segmentation models namely FPN, U-Net, and PSPNet from which LinkNet-34 model has outperformed best.
- Further, the performance of EfficientNetB7 used as encoder in LinkNet-34 model has been compared with three encoders namely ResNet34, MobileNet_V2, and ResNet50 to learn complex and abstract features at different levels of abstraction to capture information about edges, textures, shapes, and other patterns present in the input image.
- The proposed model is optimized using three different optimizers such as RMSProp, Adamax and Adam to adjust the model's internal parameters iteratively during training, aiming to minimize the loss function. The choice of optimizer and its hyperparameters can significantly impact the model's performance and training speed, making it an essential aspect of the training process.

## Proposed methodology

**Figure 1:**
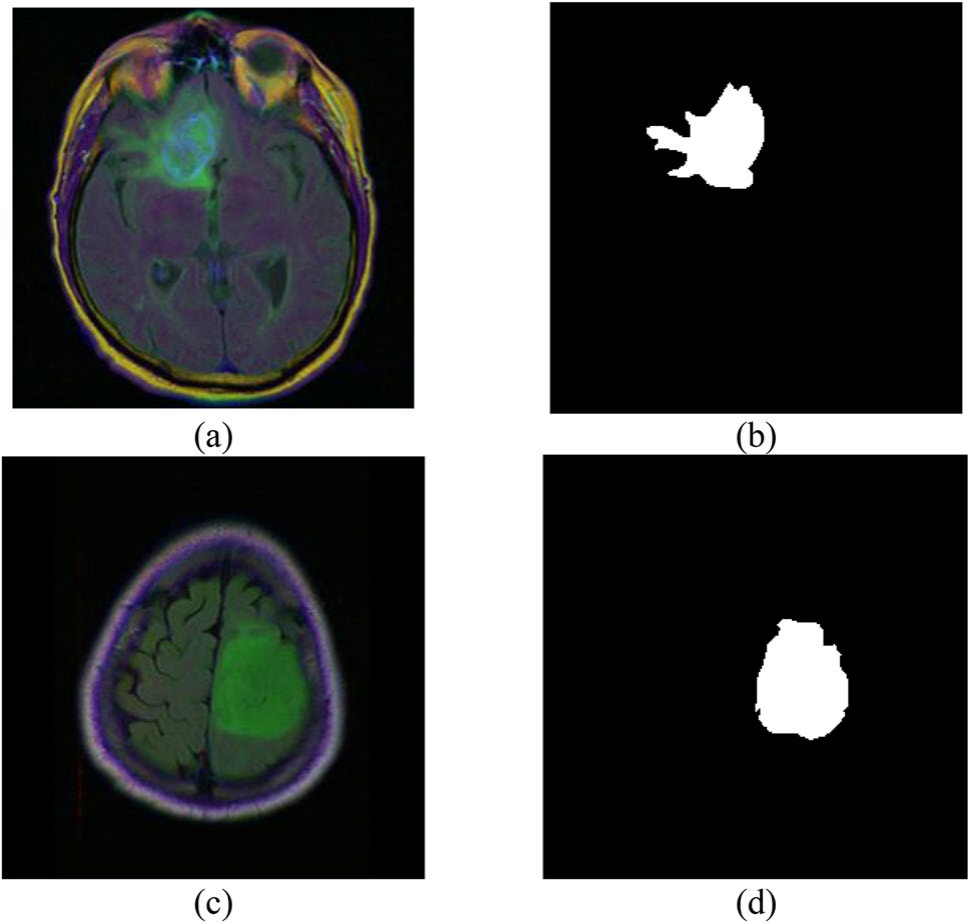
Dataset samples (**a**) original image 1, (**b**) image 1 mask, (**c**) original image 2, (**d**) image 2 mask.

Here, encoder–decoder based architecture with deep convolutional neural network is used for semantic segmentation of tumor in brain MRI images. Here, two datasets are used for model simulation where, the first brain MRI dataset includes 3929 brain MRI images of tumor from 110 patients with FLAIR anomalies for testing and training. Figure 1 shows the brain tumor MRI images^31,32^ taken for the proposed methodology. The dataset is split into 80:20 ratios where, 80% of the data is used for training and the remaining 20% is used for testing. Figure 1a and c shows the original image 1 and original image 2 respectively. Figure 1b and d displays the image 1 mask and image 2 mask respectively.

**Figure 2:**
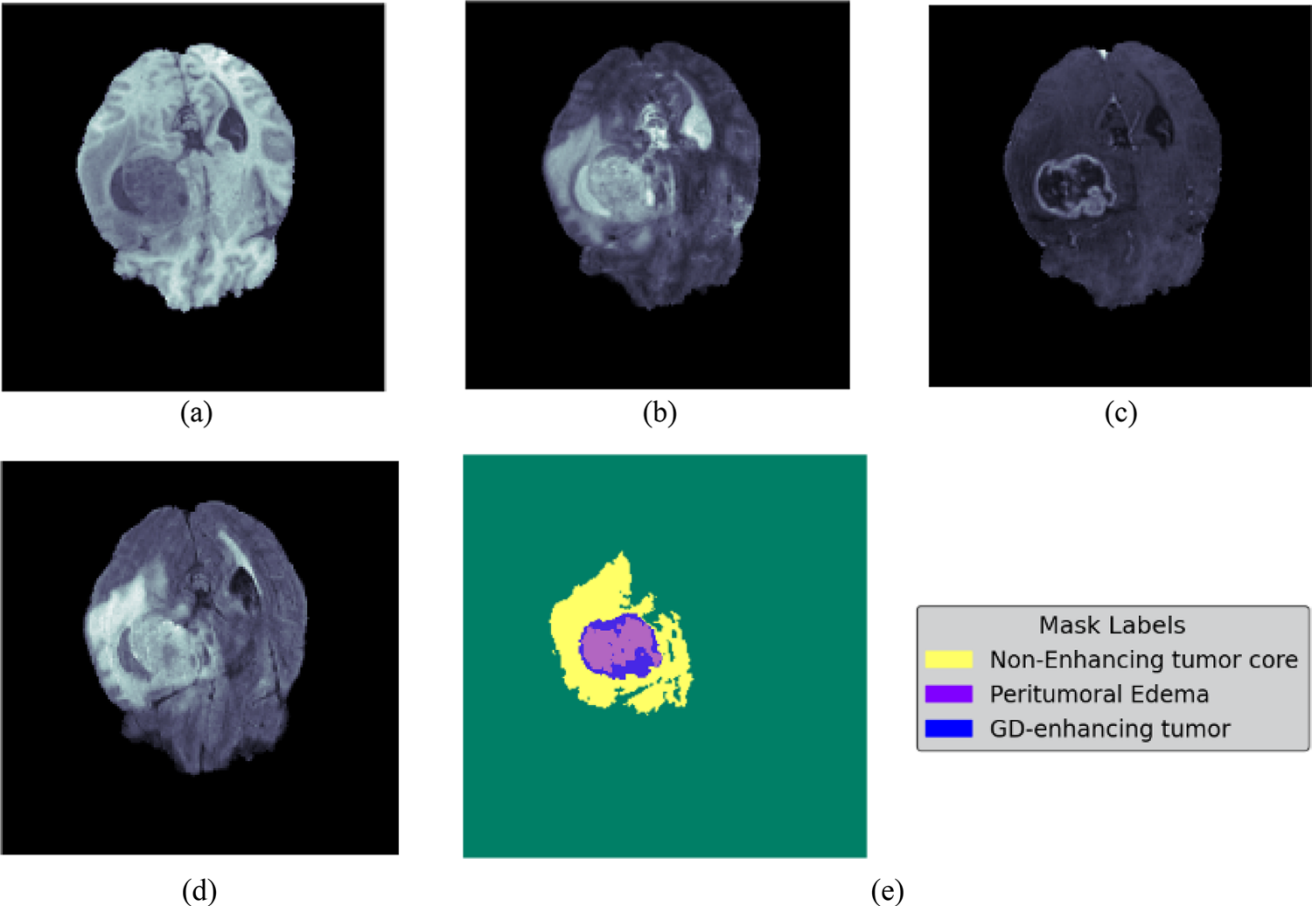
BraTs2020 samples (**a**) T1, (**b**) T2, (**c**) T1CE, (**d**) flair, (**e**) Groundtruth mask.

The second dataset considered is BraTs2020 dataset containing all four modalities T1, T1 Contrast Enhancement (CE), T2, and FLAIR along with corresponding segmentation masks. Figure 2a shows the T1 image, b shows T2 image, c shows T1CE image, d shows Flair, and e shows Groundtruth mask.

**Figure 3:**
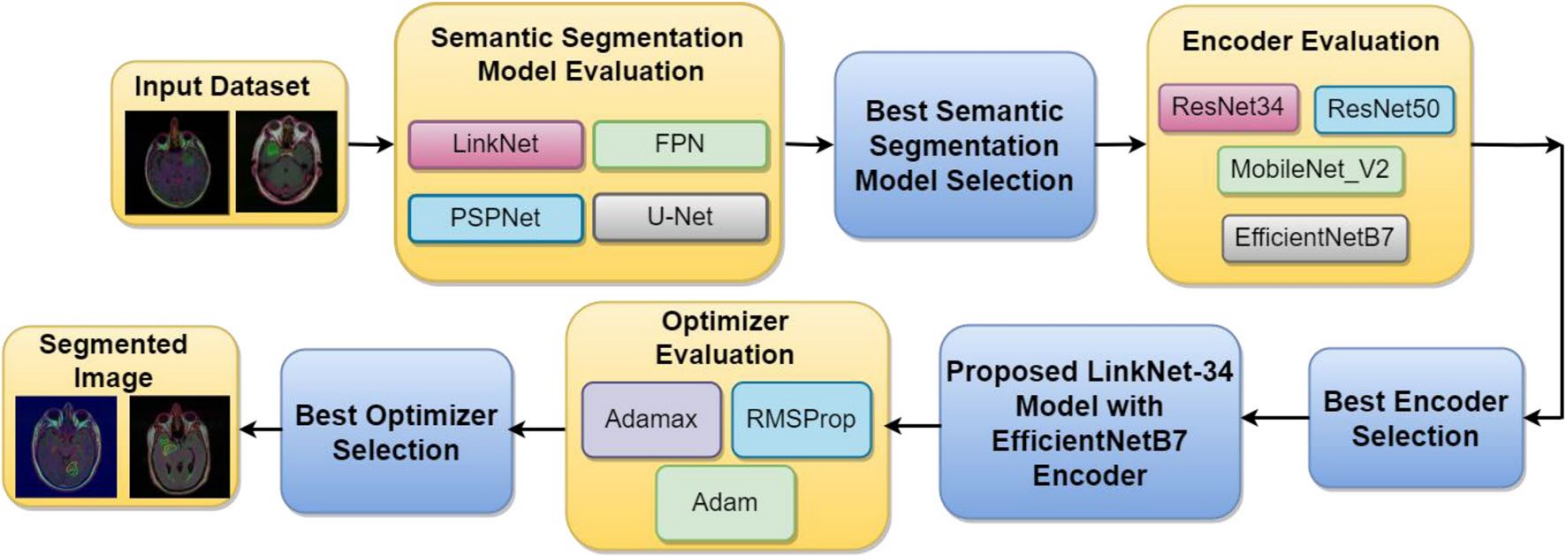
Proposed methodology for segmentation of brain tumor from MRI images.

To do pixel-wise prediction, the encoder first applies a series of filters and pooling operations to the input image to extract features, while the decoder then gradually restores the encoder’s low-resolution feature maps to their original, full input resolution. The objective is to produce a high-density segmentation map of an image, in which each pixel is associated with a distinct category or type of object. The brain MRI images are applied as input to the four semantic segmentation models namely LinkNet-34, FPN, PSPNet, and U-Net for the simulation of the model. The comparison of four models is performed based on training and validation loss curves and validation mean Jaccard index and validation dice coefficient. From the comparison of four models, it is analysed that the LinkNet-34 model is performing best in terms of all performance metrics. The proposed methodology for identifying a brain tumor is depicted in Fig. 3.

When employing autoencoder neural networks for image segmentation, the encoder stage involves downsampling the input image. Learning an image's spatial and semantic properties requires the encoder component of an encoder-decoder neural network architecture. The encoder takes an image as input and uses several convolutional and pooling layers to reduce the image's resolution. Extracting the most crucial visual characteristics through downsampling helps keep the decoder's processing load to a minimum. The proposed method focuses on the image downsampling in the encoder part. For this, simulation is done using four different encoders namely ResNet34, MobileNetV2, ResNet50, and EfficientNetB7 on the LinkNet-34 architecture to determine which encoder performs best. After the simulations, the EfficientNetB7 encoder is obtained as the best-performing encoder on the best-performing LinkNet-34 model.

From the experimental analysis, it is observed that LinkNet-34 model has performed as the best semantic segmentation model in comparison to FPN, PSPNet, and U-Net models. After that, four different encoders namely ResNet34, MobileNetV2, ResNet50, and EfficientNetB7 are applied to the best LinkNet-34 model where EfficientNetB7 has performed best as an encoder. Based on the results, LinkNet-34 model with EfficientNetB7 encoder is proposed to segment tumor from brain MRI images.

Lastly, the proposed model is optimized using three different optimizers namely RMSProp, Adam and Adamax by adjusting the model's parameters to minimize the loss function efficiently. The choice of optimizer and its hyperparameters can significantly impact the model's performance and training speed, making it an essential aspect of the training process. The best optimizer obtained from the three optimizers is the Adamax optimizer. With the help of an optimized LinkNet-34 model and EfficientNetB7 encoder, segmented images of the brain are obtained.

## Results and discussion

Here, an intelligent LinkNet-34 model with EfficientNetB7 encoder based semantic segmentation model is proposed. The performance of LinkNet-34 model is compared with other three models namely FPN, U-Net, and PSPNet. In addition, the performance of the EfficientNetB7 employed as an encoder in the LinkNet-34 model has been compared to three other encoders: ResNet34, MobileNet_V2, and ResNet50. Subsequently, the proposed approach is enhanced through the use of three distinct optimizers, namely RMSProp, Adamax, and Adam.

### Semantic segmentation model evaluation

The brain MRI scans are utilized as input for each of the four semantic segmentation models. The simulation of the model involves the use of four semantic segmentation models: LinkNet-34, FPN, PSPNet, and U-Net. These four models are used to analyze a brain MRI dataset consisting of 3929 images of brain tumors. A comparison of four models is conducted using training and validation loss curves, as well as the validation mean, Jaccard index and the validation dice coefficient.

#### LinkNet-34 Model Evaluation

The LinkNet-34 model is employed in this section to perform semantic segmentation on brain images, specifically targeting tumor detection. The distinguishing characteristic of LinkNet-34 is its ability to surpass previous semantic segmentation systems in terms of efficiency, while still maintaining a comparable level of accuracy. LinkNet-34 may be applied to several semantic segmentation tasks, including scene parsing, object recognition, etc. PyTorch and TensorFlow, which are widely used deep learning frameworks, can be utilized for their implementation.

**Figure 4:**
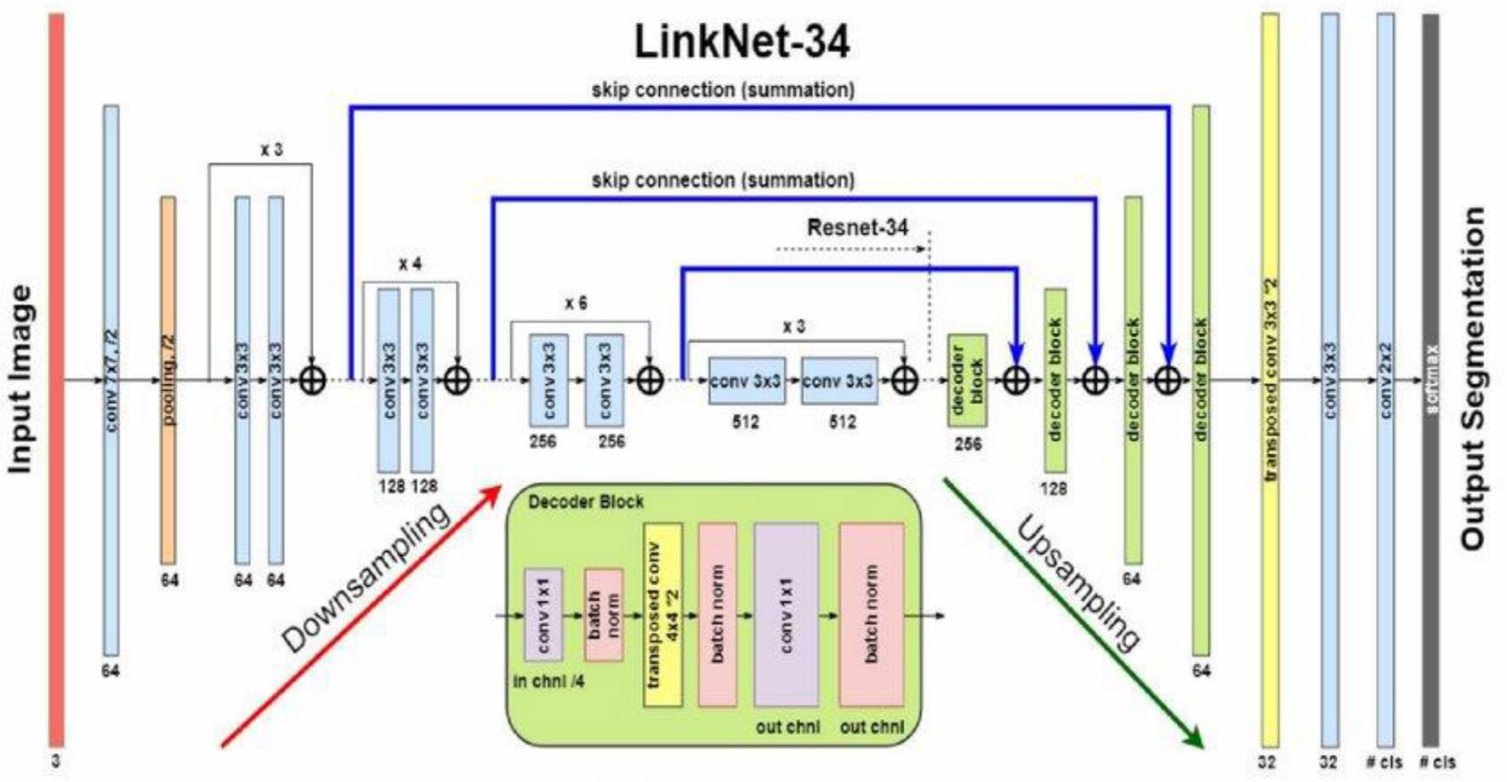
LinkNet-34 model architecture^33^.

LinkNet-34 is a network that exclusively consists of convolutional layers, as depicted in Fig. 4. The U-Net architecture, renowned for its effectiveness in semantic segmentation tasks, serves as the foundation for this approach. It has the capability to partition a diverse range of objects, encompassing objects seen in medical imaging, and satellite images. LinkNet-34 is a type of architecture that combines an encoder and a decoder to enhance the training process of segmentation models, with the goal of making it more streamlined and effective. It consists of an encoder network that downsamples the input image to capture high-level features and a decoder network that upsamples the feature map to generate pixel-level predictions. LinkNet-34 uses skip connections to connect the encoder and decoder. Utilizing skip connections, similar to U-Net, helps to propagate low-level features directly to the decoder and combine them with high-level features.

**Figure 5:**
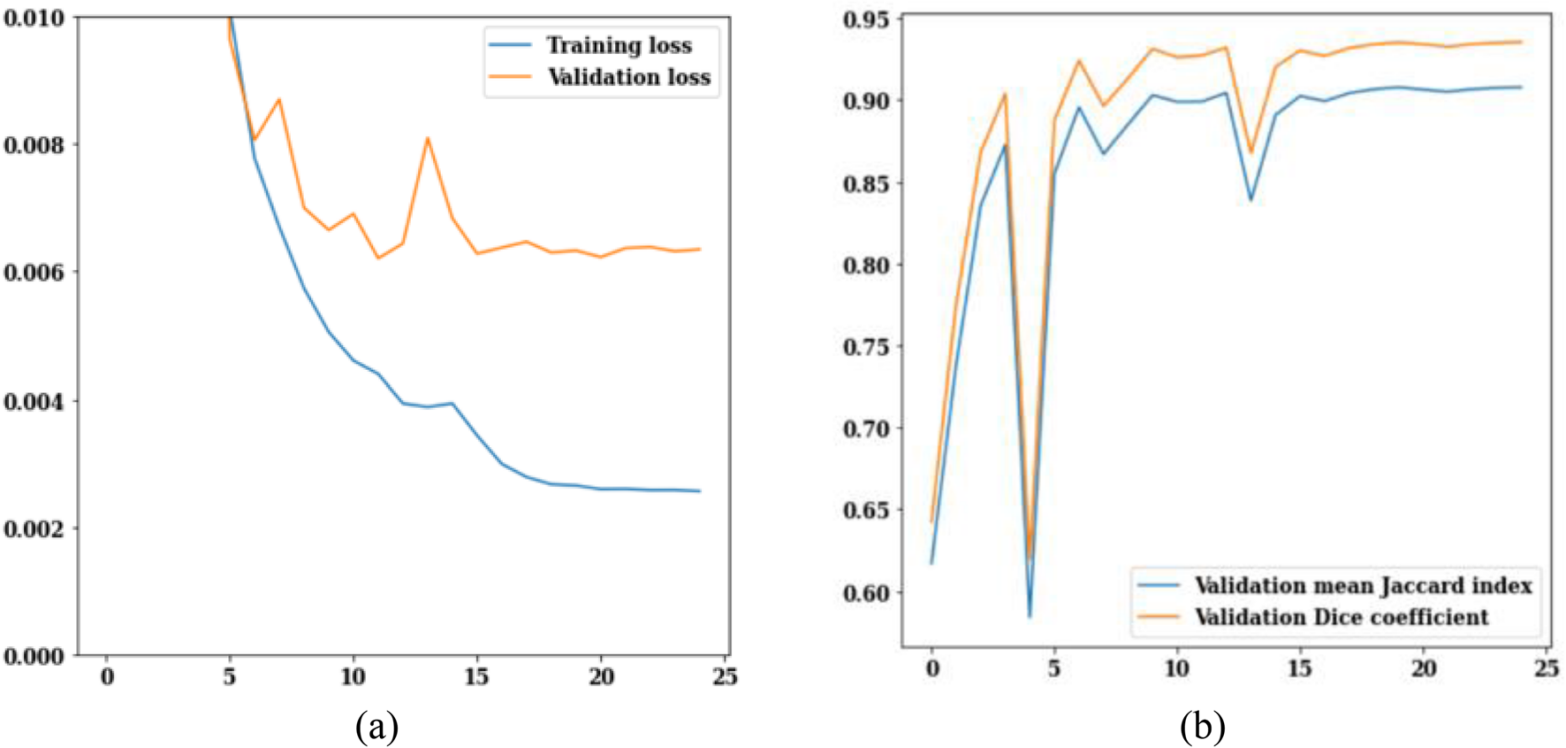
LinkNet-34 training and validation curves (**a**) loss, (**b**) mean Jaccard Index and dice coefficient.

The LinkNet-34 model has been applied to a dataset of 3929 MRI scans of brain tumors. LinkNet-34 model architecture's training and validation curves are depicted in Fig. 5. The values of the training and validation losses throughout 25 epochs are displayed in Fig. 5a. The curve analysis reveals a loss value range of 0.002–0.010. Validation loss is 0.008 on the 13th epoch and 0.006 on the 25th epoch, respectively. If this trend is generalized, it can be seen that as epoch increases, loss decreases.

Figure 5b shows the validation curves for Jaccard index and dice coefficient. There is a strong correlation between the Jaccard index and the dice coefficient. In actuality, the model rankings obtained from the two metrics are same under all circumstances. The Jaccard index and dice coefficient both have a value of 0.6 on the 5th epoch. However, on the 25th epoch, the validation dice coefficient is 0.94 and the validation mean Jaccard index is 0.90, both are increased from earlier epochs. From the Fig. 5, it can be concluded that, LinkNet-34 architecture is showing the value of validation dice coefficient as 0.94 and validation mean Jaccard index as 0.90 respectively.

#### FPN model evaluation

**Figure 6:**
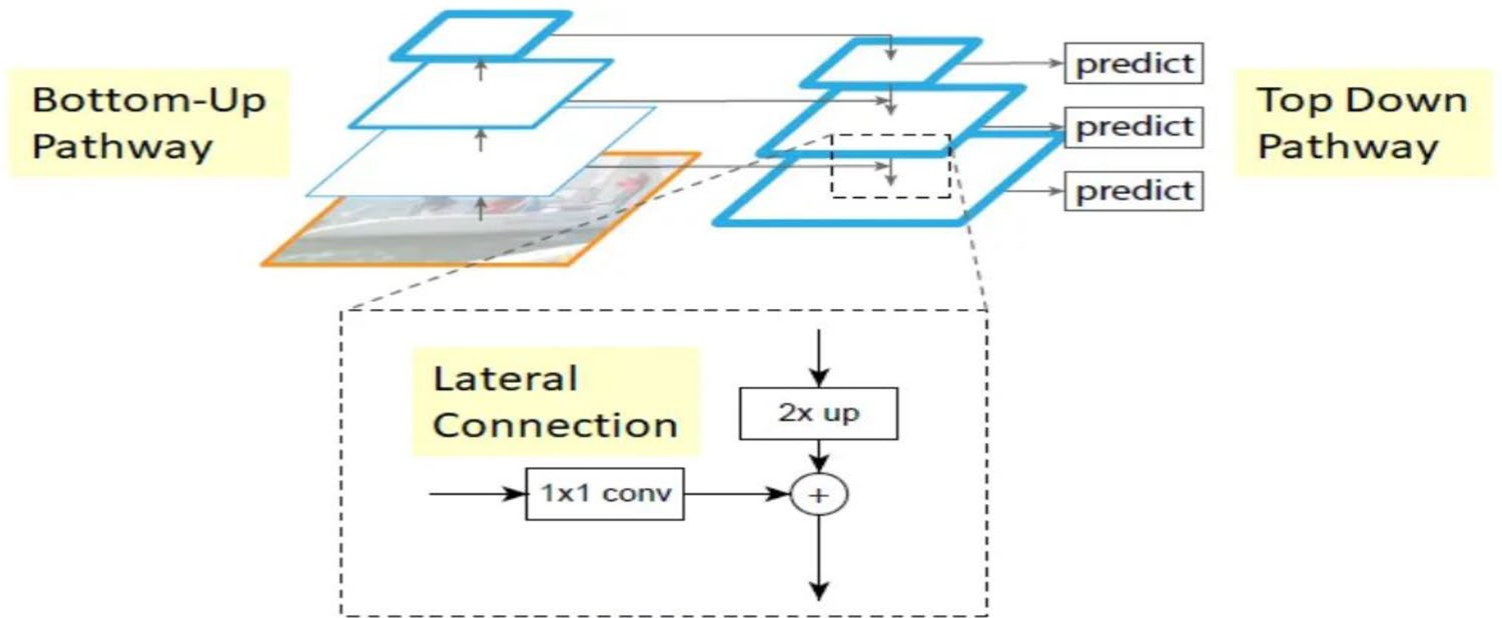
FPN model architecture^34^.

In this section, the FPN, which stands for “Feature Pyramid Network,” is used for the segmentation purpose. It is designed to address the problem of scale variation in object detection tasks. It introduces a top-down pathway to the traditional convolutional neural network, allowing the model to fuse high-level semantic information with multi-scale features at different resolutions. Primarily employed in object detection frameworks, where it enhances the ability of the detector to handle objects of different sizes. The architecture consists of two pathways. First is Bottom-up pathway (Normal feed-forward CNN) and the second is Top-down pathway (New architecture used for merging features). Figure 6 represents FPN model architecture.

The feedforward processing of the central ConvNet is the bottom-up pathway. Each stage corresponds to one “level” of the pyramid. Top-down pathway enrichment through lateral connection will use the output of the final layer of each stage as a reference set of mappings of features. In order to lower the channel dimensions, the bottom-up pathway's feature maps are convolved 1:1.

For convenience, the nearest neighbour is used to upsample the higher resolution features by a factor of 2 in Top-Down Pathway. Each lateral link combines feature maps from the bottom-up and top-down directions with the same spatial resolution. Element-wise addition is used to combine the bottom-up and top-down feature maps.

**Figure 7:**
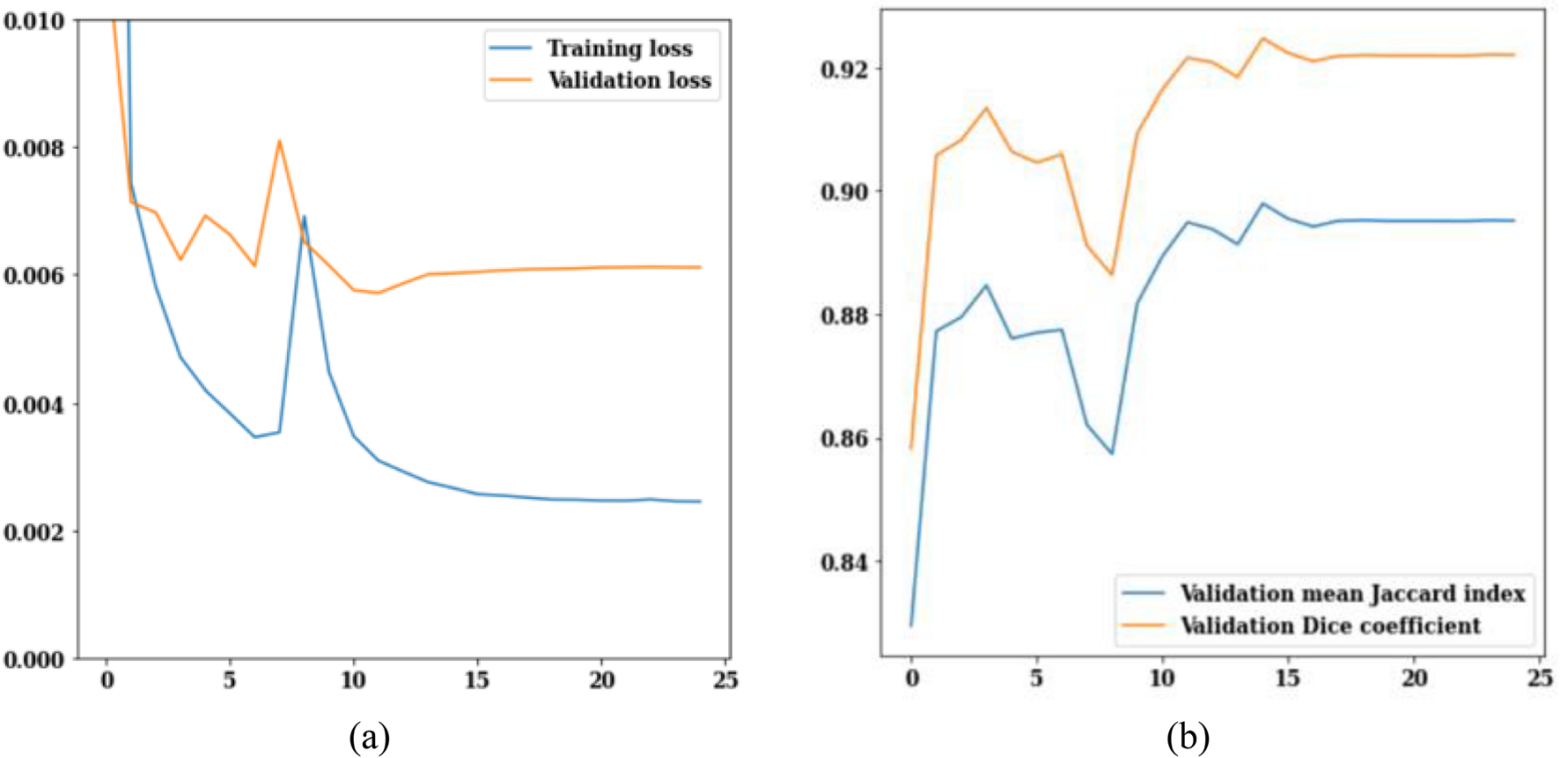
FPN training and validation curves (**a**) loss, (**b**) mean Jaccard Index and dice coefficient.

FPN model has been implemented on the brain tumor dataset that consists of 3929 MRI images. Figure 7 displays the FPN model's training and validation curves. Figure 7a depicts training and validation loss from which it can be analysed that on the 25th epoch, the value of validation loss is 0.006 and training loss is 0.002 and both are decreasing with increasing epochs. Validation Jaccard index and dice coefficient values are displayed in Fig. 7b. Dice coefficient on the 3rd epoch is 0.91, and the Jaccard index is 0.88. On the 25th epoch, the highest possible Jaccard index is 0.89, while the highest possible dice coefficient is 0.92. It can be concluded from Fig. 6 that, the value of validation loss, Jaccard index and dice coefficient at 25th epoch is 0.006, 0.89 and 0.92 respectively.

#### PSPNet model evaluation

**Figure 8:**
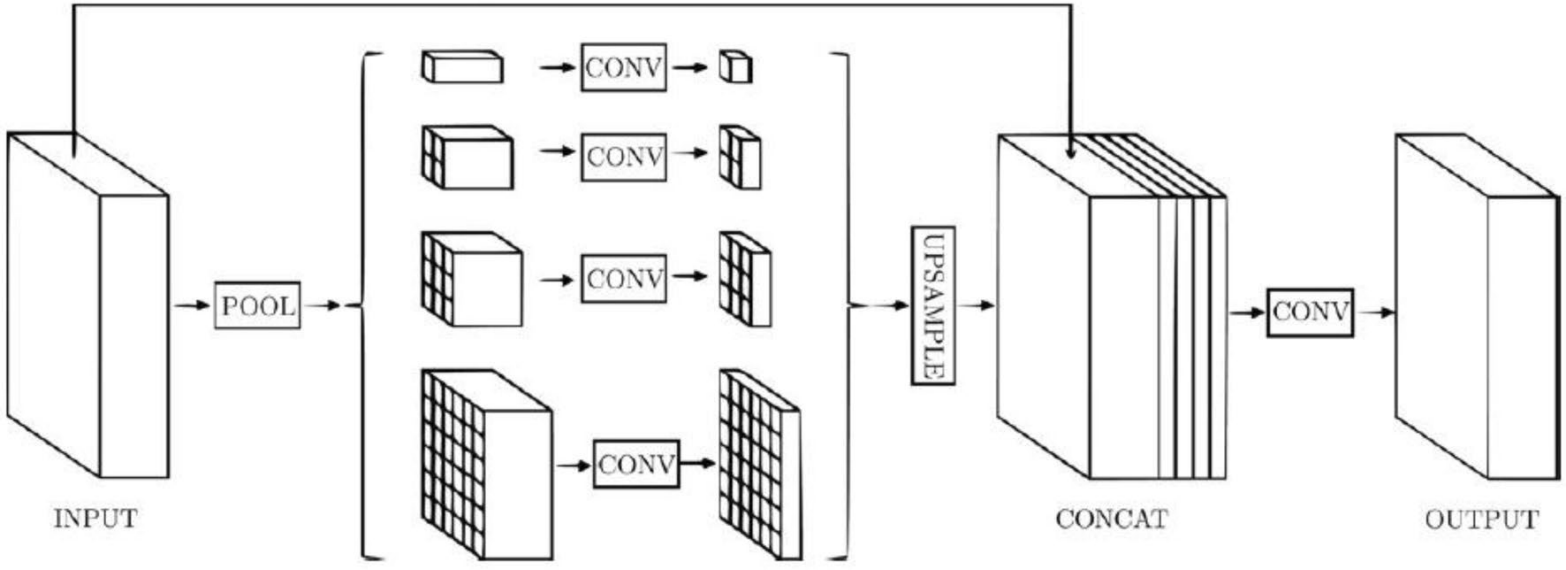
PSPNet model architecture^35^.

Semantic segmentation tasks are where PSPNet really shines. Semantic segmentation aims to divide an input image into regions belonging to different item categories by assigning a semantic label to every pixel in the image. PSPNet uses pyramid pooling modules to capture multi-scale context information from different regions of the input image as shown in Fig. 8. This helps the model make more informed pixel-wise predictions, especially for objects of varying sizes. PSPNet adopts a pyramid structure, where the input feature map is downsampled multiple times, and global pooling is applied at different scales to capture contextual information. The features are then upsampled and combined to make pixel-level predictions. PSPNet uses global pooling at different pyramid levels to capture context, but it lacks the explicit feature fusion step that FPN employs. Instead, it uses convolutional layers for upsampling and feature combination. PSPNet is well-suited for tasks where pixel-level segmentation is essential, such as scene parsing, image segmentation, and fine-grained object recognition.

**Figure 9:**
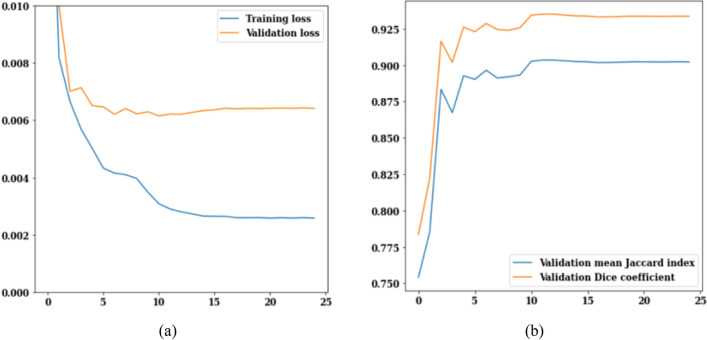
PSPNet training and validation curves (**a**) loss, (**b**) mean Jaccard Index and dice coefficient.

The PSPNet model has been applied to a dataset of 3929 MRI scans of brain tumors. Figure 8 shows the loss, Jaccard index, and dice coefficient curves over training and validation. The values of training and validation loss over 25 epochs are shown in Fig. 9a. Loss is expected to go down from its current values of 0.010 for validation loss and 0.002 for training loss. The values of the Jaccard and dice coefficients for 25 epochs are displayed in Fig. 9b. Dice coefficient on the 3rd epoch is 0.91 and Jaccard index is 0.875. On the 25th epoch, the values for both the Jaccard index and the dice coefficient peaked at 0.925 and 0.900, respectively. Figure 9 summarizes the data, revealing that on the 25th epoch the training loss is 0.002, the Jaccard index is 0.925, and the dice coefficient is 0.900.

#### U-Net model evaluation

In this section, the U-Net model evaluation is performed to obtain the values of loss, jaccard index and dice coefficient values on 25 epochs. The U-Net model consists of three components such as contracting path, expanding path and bottleneck^36,37^.

**Figure 10:**
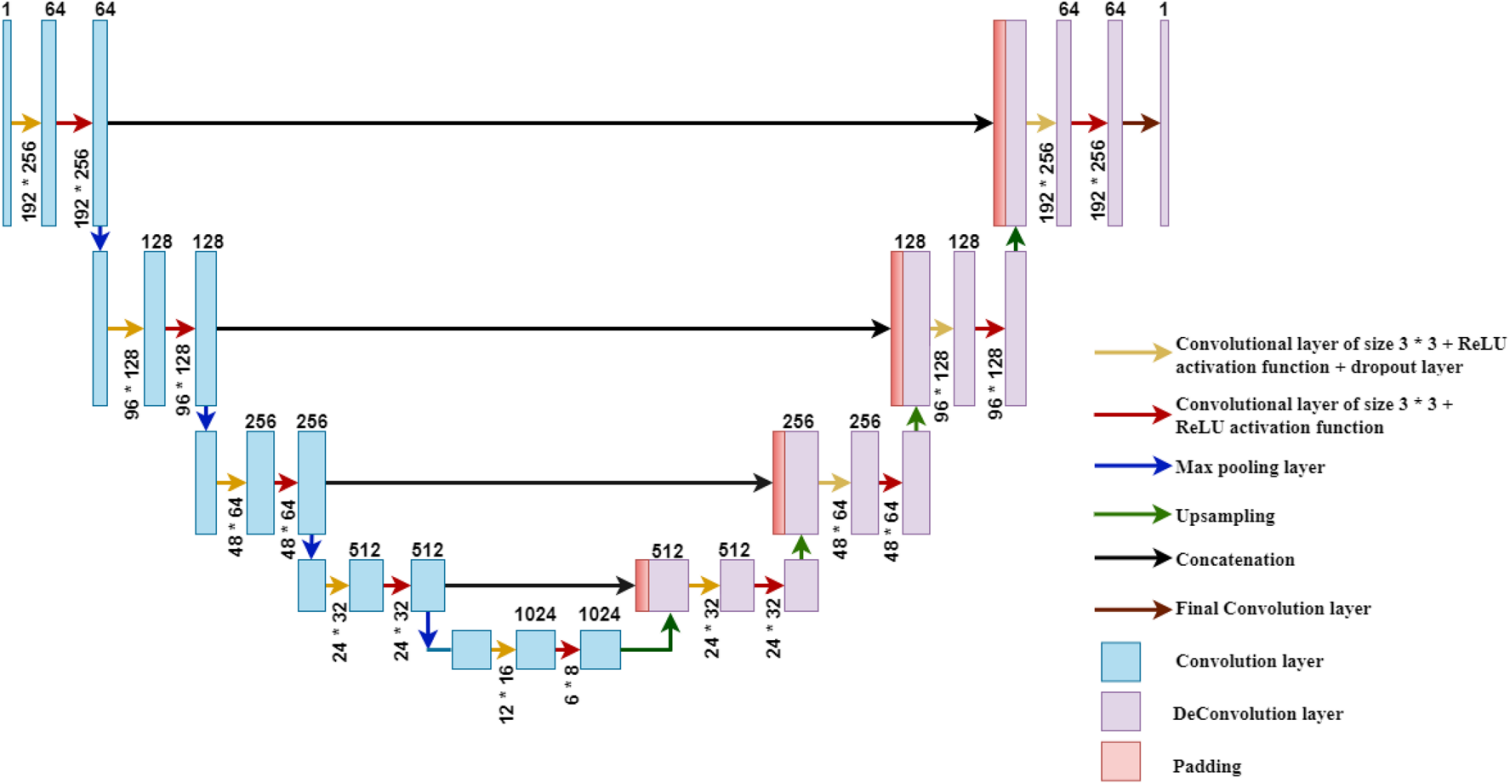
U-Net model architecture^38^.

U-Net is an encoding–decoding system. There is an encoding section (the encoder) and a decoding section (the decoder). During encoding an image, the input is downsampled so that high-level information may be captured; during decoding, the image is upsampled so that predictions can be made at the pixel level. U-Net's skip links between the encoder and decoder allow it to pick up contextual information. U-Net is able to do this by combining coarse-grained data from the encoder with upsampled features from the decoder. U-Net shines when working with small objects and sparse training data. Successful applications include cell and organ segmentation in medical images and other biomedical image segmentation tasks. Figure 10 represents the broad U-Net model architecture.

**Figure 11:**
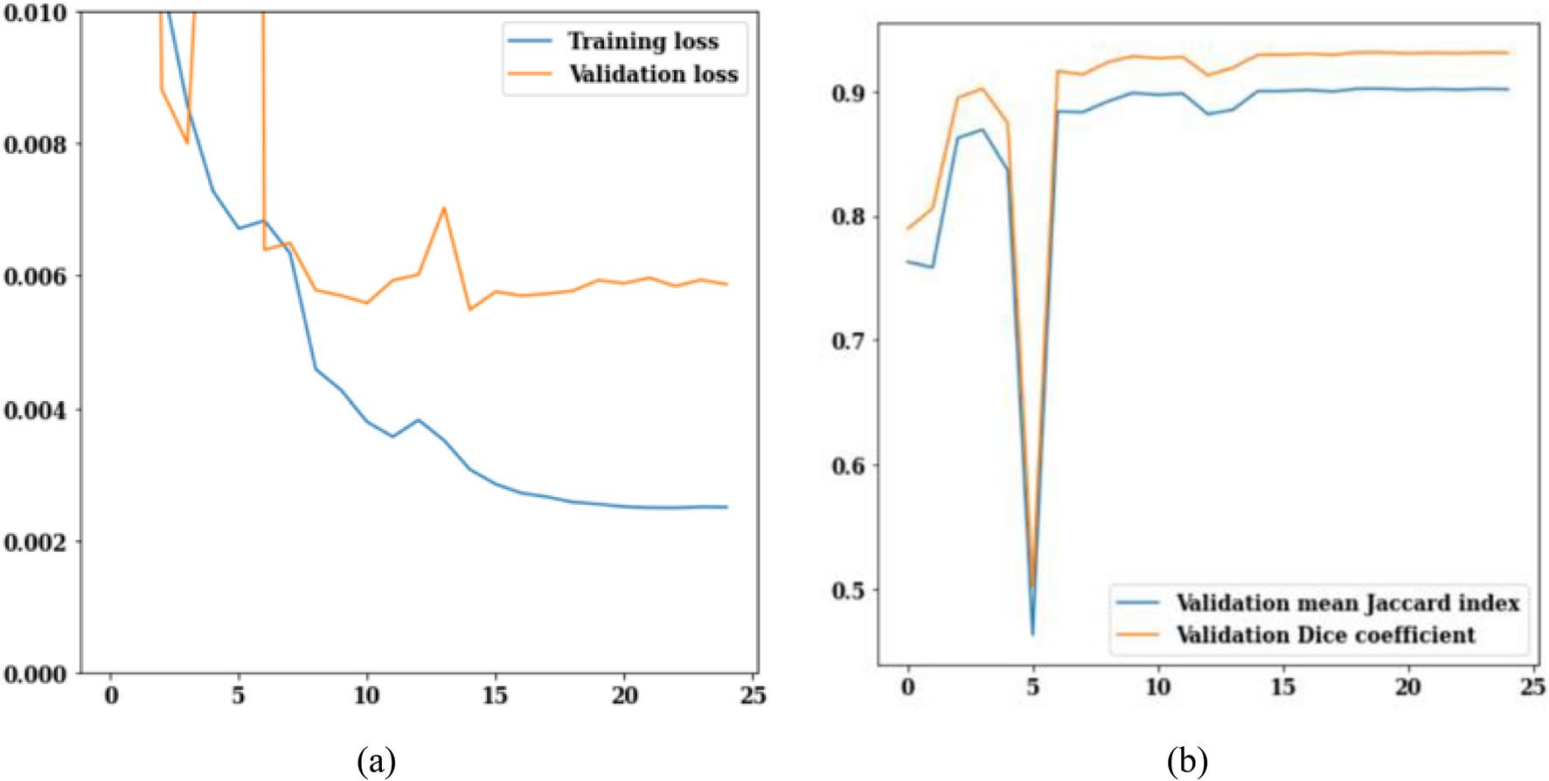
U-Net training and validation curves (**a**) loss, (**b**) mean Jaccard Index and dice coefficient.

U-Net model has been implemented on the brain tumor dataset consisting of 3929 MRI images. Figure 11 displays the U-Net model's training and validation curves. The U-Net model loss curves are depicted in Fig. 11a. As the epoch counter increases, the loss value decreases. Training loss on the 25th epoch is 0.003, and validation loss is 0.006. The U-Net model's jaccard index and dice coefficient over 25 epochs are displayed in Fig. 11b. The validation dice coefficient is 0.9 on epoch 6, and it rises steadily with each succeeding epoch. This means that the average value of the jaccard index is 0.9 and the average value of the dice coefficient is 0.92. From Fig. 11, it can be analyzed that the validation loss is decreased to 0.006 on the 25th epoch. The values of jaccard index and dice coefficient on the 25th epoch are 0.9 and 0.92 respectively.

### Best semantic segmentation model selection

**Figure 12:**
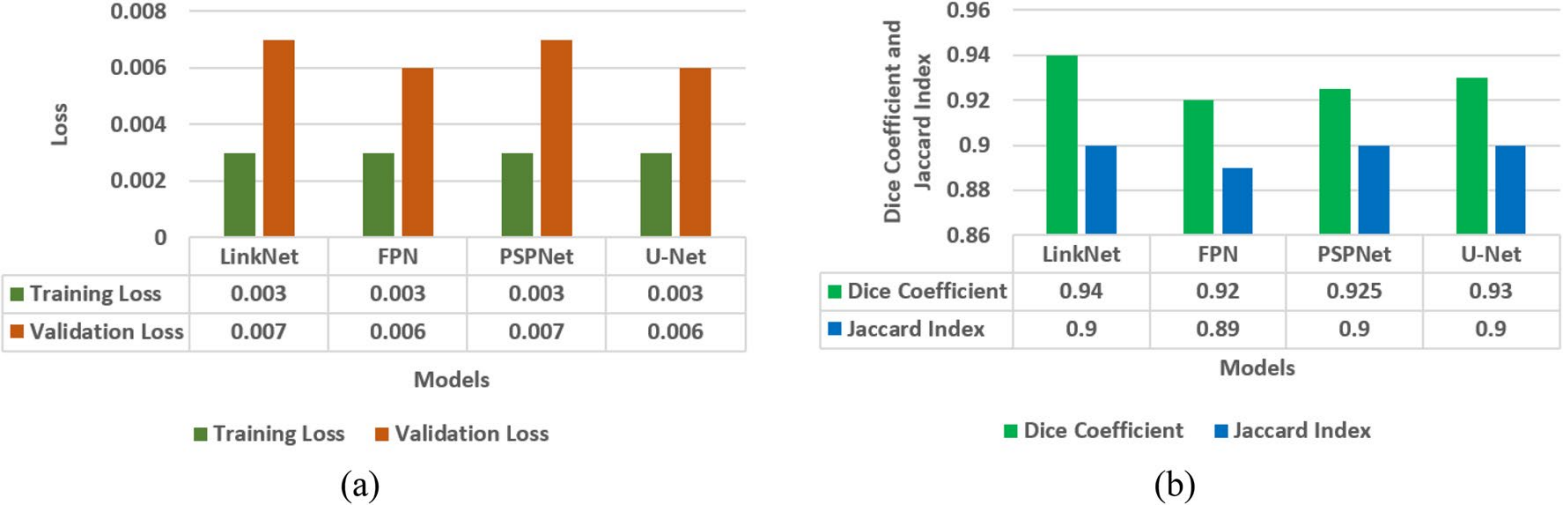
Comparison of models (**a**) training and validation loss, (**b**) dice coefficient and Jaccard Index.

In the last sections, model evaluation is performed using four models i.e. LinkNet-34, FPN, PSPNet, and U-Net. In this section, comparison of four models is performed in terms of loss analysis, jaccard index analysis and dice coefficient analysis. Figure 12 shows the results of a loss analysis, jaccard index analysis, and a comparison of models using the dice coefficient. Using the Dice coefficient and the Jaccard index, Fig. 12b demonstrates that the dice coefficient for the LinkNet-34 model is 0.94, whereas it is 0.93 for the U-Net model. Jaccard index values of 0.9 are shared by all three models namely LinkNet-34, PSPNet, and U-Net. Thus, the dice coefficient performance data are used to compare the models. Out of the four models, LinkNet-34 is the most successful in terms of dice coefficient. Four models' training and validation loss information for comparison are displayed in Fig. 12a. Validation losses of 0.007 are achieved by LinkNet-34 and PSPNet, while those of 0.006 are achieved by FPN and U-Net.

From the Fig. 12a it can be concluded that LinkNet-34 model is showing the highest value of dice coefficient as 0.94. Although the value of Jaccard index is 0.9 on LinkNet-34, PSPNet and U-Net models. Whereas, the value of loss is approximately same on all the four models.

### Encoder evaluation for best semantic segmentation model

In semantic segmentation, the encoder is a crucial component responsible for downsampling the input image while extracting meaningful features. The downsampling process is essential for reducing the spatial dimensions of the input image to create a feature map that captures high-level semantic information and spatial context. The encoder accomplishes this through a series of convolutional and pooling layers.

From the last section, it can be concluded that LinkNet-34 model has performed as the best semantic segmentation model in comparison to FPN, PSPNet, and U-Net models. In this section, four different encoders namely ResNet34, MobileNetV2, ResNet50, and EfficientNetB7 are applied to the best LinkNet-34 model to get the best encoder. It helps to transform the input image into a more compact and abstract representation, where each pixel in the feature map corresponds to a higher-level semantic concept rather than pixel-level information. In order to generate predictions for the semantic class of each pixel in the final segmentation map, the decoder uses this abstract representation.

#### ResNet34 encoder evaluation

In this section, ResNet34 is used as an encoder in the LinkNet-34 model. ResNet34 is a common deep CNN architecture used for image classification because of its high layers count as 34. ResNet34 uses a method called residual connections to deal with the vanishing gradient problem. Residual links are those that go across the network but not all of its tiers. The speed with which ResNet34 can be taught and the accuracy with which it can generate predictions make it a particularly efficient architecture.

**Figure 13:**
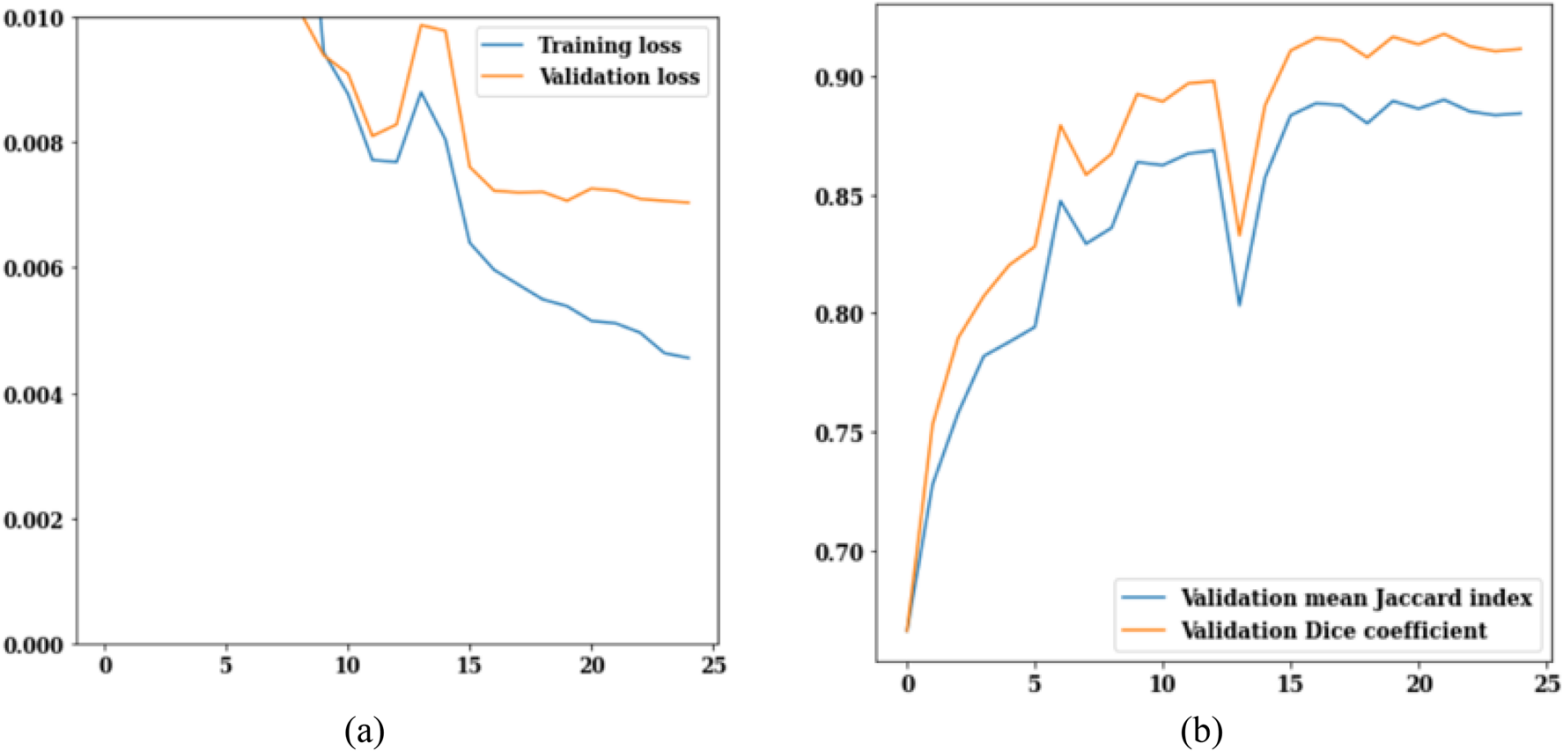
ResNet34 training and validation curves (**a**) loss, (**b**) mean Jaccard Index and dice coefficient.

The loss, Jaccard index, and dice coefficient graph for ResNet34 are displayed in Fig. 8. Loss values during 25 epochs with the ResNet34 model are displayed in Fig. 13a. After the 12th epoch, training loss begins to decrease, reaching a low of 0.005 by the 25th epoch. After twenty-five iterations, the validation loss is at 0.007. The Jaccard index and dice coefficient values for 25 epochs are displayed in Fig. 13b. Validation mean Jaccard index is 0.88 and validation dice coefficient is 0.91 in the 25th epoch. To sum up Fig. 13, it can be concluded that the validation loss is decreased to 0.007 and the values of validation mean Jaccard index and validation dice coefficient are 0.88 and 0.91.

#### MobileNetV2 encoder evaluation

In this section, MobileNetV2 is used as encoder in the LinkNet-34 model. MobileNetV2 is one of the most widely used CNN-based architectures for mobile devices due to its small size (just 53 layers) and high performance. To maximize its effectiveness, MobileNetV2 employs a method known as inverted residuals. One sort of residual connection is the inverted residual, which employs a thin bottleneck layer. The total amount of variables and processes in the inverted residual is decreased by the bottleneck layer without sacrificing accuracy. Image classification, object identification, and semantic segmentation are just some of the mobile vision tasks where MobileNetV2 has been proven to excel. In addition to its speed in both training and prediction, MobileNetV2 is a very efficient design. Squeeze-and-excitation blocks are used in the convolutional layers of MobileNetV2.

**Figure 14:**
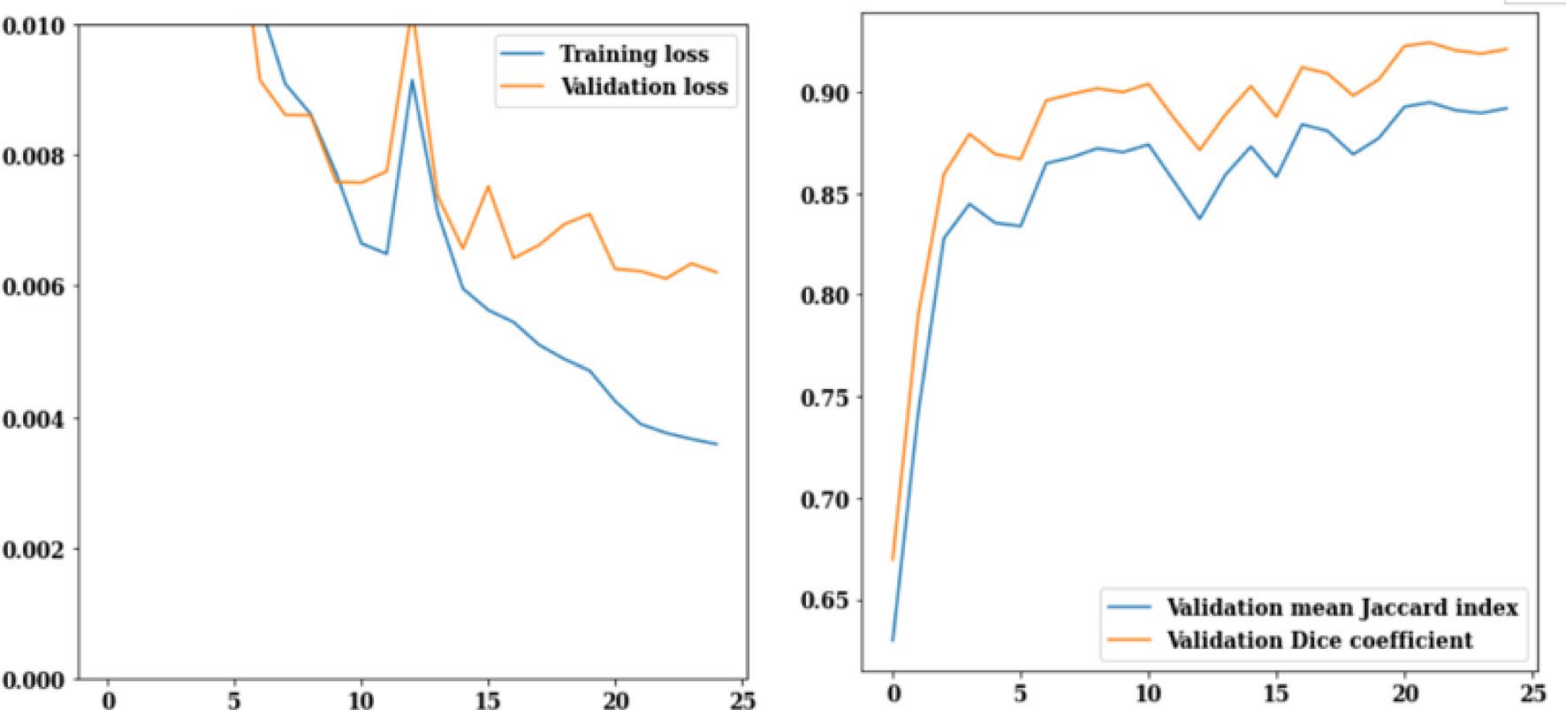
MobileNet_V2 training and validation curves (**a**) loss, (**b**) mean Jaccard Index and dice coefficient.

Loss, Jaccard index, and dice coefficient comparisons for MobileNet_V2 are displayed in Fig. 14. Figure 14a displays the MobileNet_V2 model's training and validation loss results over 25 iterations. With each passing era, the value of loss decreases. On the 25th epoch, the training loss is 0.004, whereas the validation loss is 0.006. Figure 14b displays the Jaccard index and dice coefficient values for 25 epochs. The Jaccard index and the dice coefficient both go up in value as the number of epochs goes up. The value increases sharply from the first to the third epoch, from 0.65 to 0.83. The validation mean Jaccard index for the 25th epoch is 0.87, while the validation dice coefficient is 0.92. From the Fig. 14 it can be seen that the validation loss is decreased to 0.006 and Jaccard index is 0.87, while the validation dice coefficient is 0.92.

#### ResNet50 encoder evaluation

In this section, ResNet50 is used as an encoder in the LinkNet-34 model. ResNet50 employs a method called residual connections to solve the vanishing gradient problem. When training deep CNNs, these links facilitate the gradient's smooth transit throughout the network. ResNet50 minimizes the number of network parameters by employing bottleneck layers. A bottleneck layer is one that has fewer channels than the layer above it. This allows for fewer parameters in the network to be used without sacrificing precision. To make the training process more consistent, ResNet50 employs batch normalization. The activations at each layer are normalized using a technique called batch normalization, and then the new weights are applied to the network. As a result, the network's sensitivity to data fluctuations is reduced.

**Figure 15:**
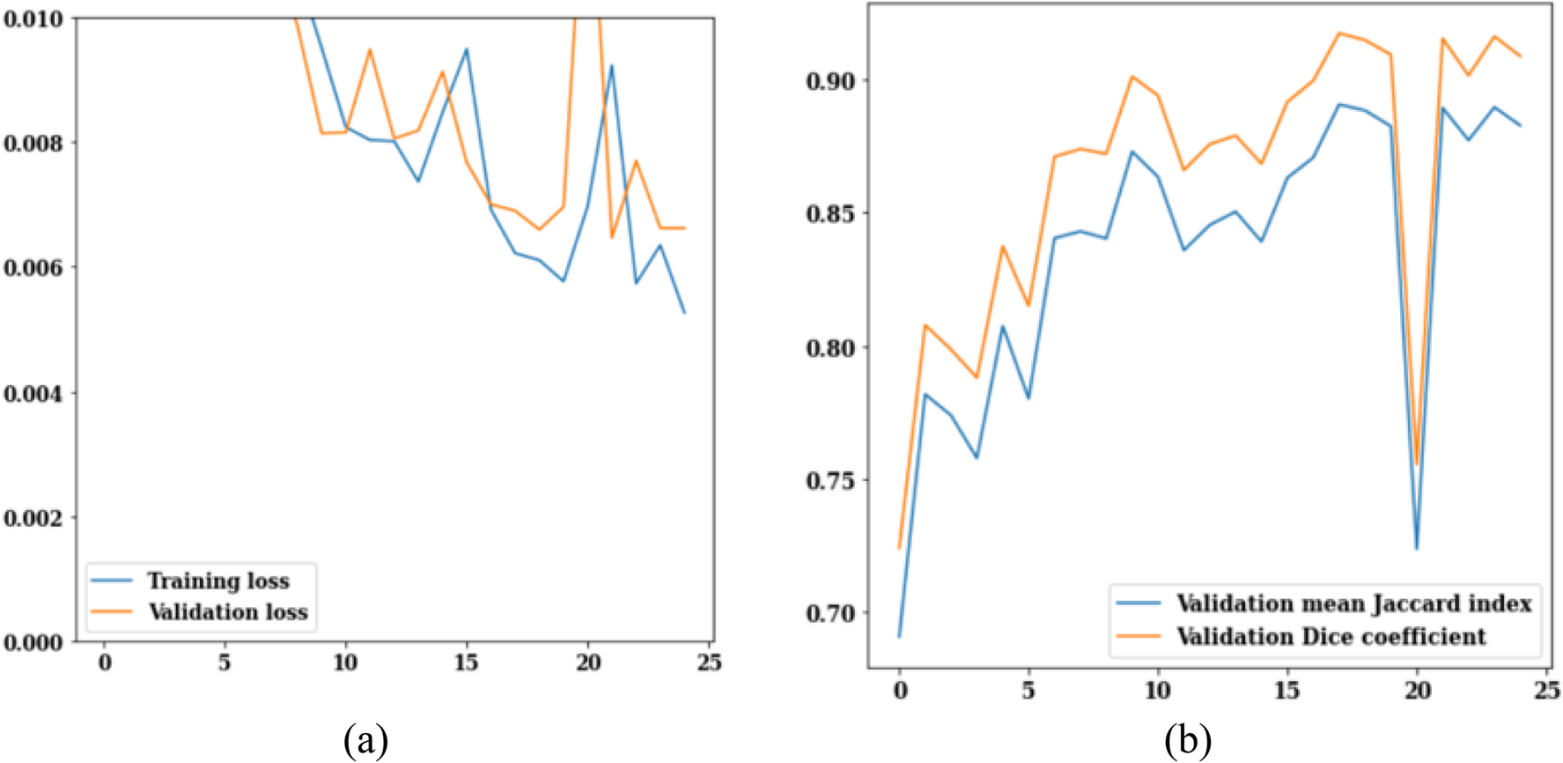
ResNet50 training and validation curves (**a**) loss, (**b**) mean Jaccard Index and dice coefficient.

In Fig. 15, the training and validation loss curves, jaccard index, and dice coefficient for ResNet50 are shown. The values of the training and validation losses throughout 25 epochs are displayed in Fig. 15a. Loss is scaled back from 0.010 to 0.006 for validation and 0.005 for training. The Jaccard index and dice coefficient are depicted in Fig. 15b. Rapid growth may be seen in the figures between the epochs of 0 and 3. The Jaccard index and dice coefficient on epoch 25 are 0.87 and 0.92 respectively. From Fig. 15, it can be concluded that the value of validation loss is 0.007 and on the 25th epoch the values of Jaccard index and dice coefficient are 0.87 and 0.92 respectively.

#### EfficientNetB7 encoder evaluation

**Figure 16:**
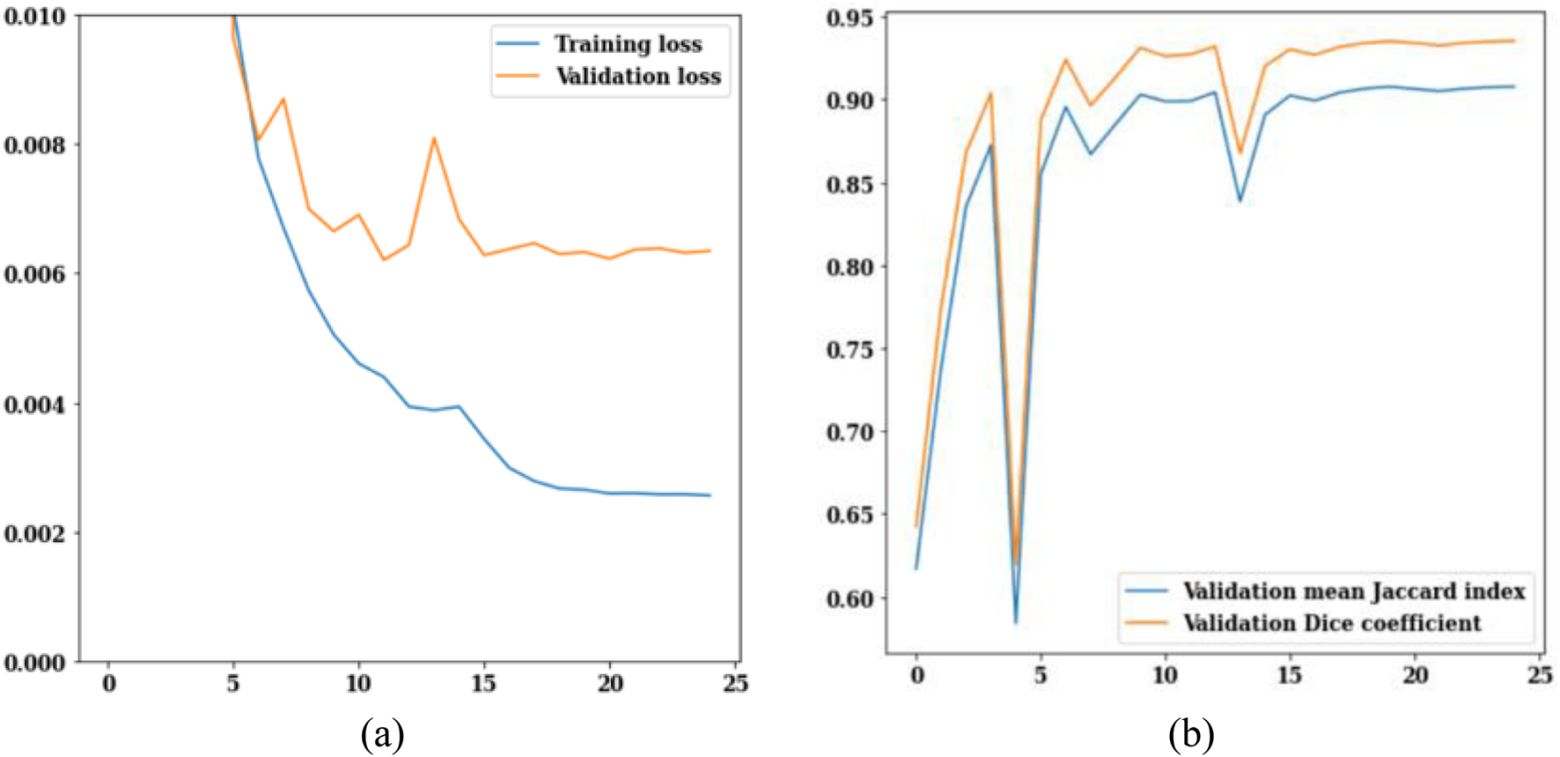
EfficientNetB7 training and validation curves (**a**) loss, (**b**) mean Jaccard Index and dice coefficient.

In this section, EfficientNetB7 encoder is applied to LinkNet-34 architecture. The EfficientNetB7 model architecture's training and validation curves are depicted in Fig. 16. The values of the training and validation losses throughout 25 epochs are displayed in Fig. 16a. The curve analysis reveals a loss value range of 0.002–0.010. Validation loss is 0.008 on the 13th epoch and 0.006 on the 25th epoch, respectively.

Figure 16b displays the validation curves for Jaccard index and dice coefficient. There is a strong correlation between the Jaccard index and the dice coefficient. In actuality, the model rankings obtained from the two metrics are same under all circumstances. The Jaccard index and dice coefficient both have a value of 0.6 on the fifth epoch, as can be observed. However, on the 25th epoch, the validation dice coefficient is 0.94 and the validation mean Jaccard index is 0.90, both of which are increased from earlier epochs. From Fig. 16, it can be concluded that the value of loss is decreased to 0.006 and the values of Jaccard index and dice coefficient are 0.90 and 0.94 respectively.

### Best encoder selection

**Figure 17:**
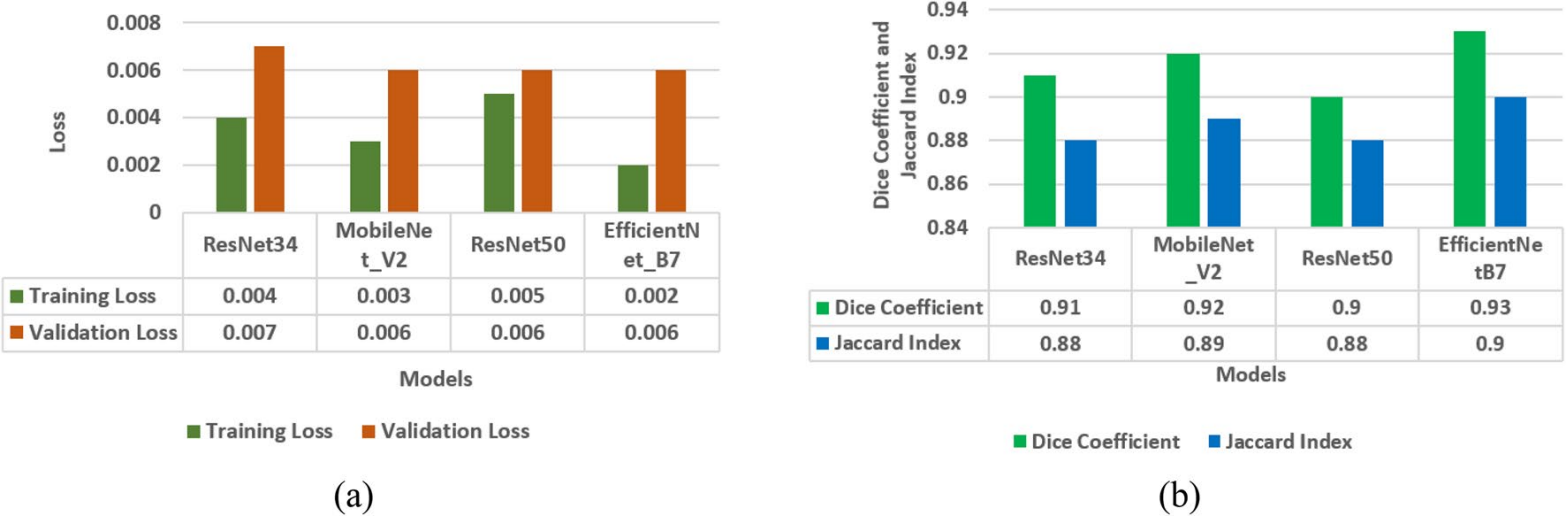
Comparison of encoders (**a**) training and validation loss, (**b**) mean Jaccard Index and dice coefficient.

In the last sections, four different encoders namely ResNet34, MobileNetV2, ResNet50, and EfficientNetB7 are applied on the LinkNet-34 architecture for the segmentation of tumor in brain MRI images. Encoders are compared in terms of dice coefficient, Jaccard index, and loss as shown in Fig. 17.

The four encoders' training and validation loss values are displayed in Fig. 17a. Training loss for the EfficientNetB7 encoder is just 0.002, and validation loss is 0.006 which is least as compared to other three encoders. The four encoders' dice coefficient and Jaccard index values are displayed in Fig. 17b. From this figure, it can be seen that dice coefficients are 0.91, 0.92, 0.90, 0.93 and Jaccard index are 0.88, 0.89, 0.88, 0.90 for ResNet34, MobileNetV2, ResNet50 and EfficientNetB7 encoder respectively. From these results, it is analysed that EfficientNetB7 encoder has outperformed best in terms of dice coefficient and Jaccard index as 0.94 and 0.90 respectively. As a result, EfficientNetB7 might be considered the best-performing and most successful encoder that enables downsampling process in the LinkNet-34 semantic segmentation model to efficiently process and understand the spatial context and high-level features, making it capable of accurate pixel-wise semantic segmentation.

### Proposed LinkNet-34 model with EfficientNetB7 as encoder

From the previous discussion, it is analysed that segmentation results are coming best with the combination of LinkNet-34 with EfficientNetB7 as encoder. Hence here, LinkNet-34 with EfficinetNetB7 model is proposed for the segmentation of brain tumour in MRI images. It combines the efficiency of EfficientNetB7 with the detailed segmentation capabilities of LinkNet-34, resulting in a high-performing and computationally efficient model for semantic image segmentation. The architecture can be adapted and fine-tuned for various semantic segmentation tasks, making it a versatile choice for researchers and practitioners working on segmentation tasks in biomedical images.

The LinkNet-34 design utilizes EfficientNetB7 as the encoder to extract high-level information from the input image through the application of deep convolutional neural networks. The efficient architecture of EfficientNetB7 enables it to effectively collect significant image features. The EfficientNetB7 encoder is constructed using the MBConv (Mobile Inverted Residual Bottleneck) block as its fundamental building unit. A depthwise separable convolution decomposes a conventional convolution into two sequential operations: a depthwise convolution and a pointwise convolution. The depthwise separable convolution technique can be mathematically described by Eq. (1) when applied to the input tensor X:

1 $$ {\text{DepthwiseConv(X)}} = {\text{PointwiseConv}}({\text{DepthwiseConv}}_{1} ({\text{X}})) $$

where DepthwiseConv1 characterizes the depthwise convolution operation and PointwiseConv indicates the pointwise (1 × 1) convolution operation.

The Squeeze-and-Excitation (SE) module adjusts channel-specific feature responses. The SE block method can be described by Eq. (2) using the input tensor X:

2 $$ {\text{SE(X)}} = {\text{Sigmoid}}({\text{FullyConnected}}({\text{GlobalAveragePooling}}({\text{X}}))) $$

The GlobalAveragePooling operation calculates the mean value for each channel. The FullyConnected layer represents a dense layer and Sigmoid function is used as the activation function.

The EfficientNetB7 encoder is constructed by stacking multiple MBConv blocks with different expansion ratios, kernel sizes, and output channel sizes. The overall encoder operation E(X) for an input tensor X in EfficientNetB7 can be represented as a sequence of MBConv blocks as shown in Eq. (3):

3 $$ {\text{E}}({\text{X}}) = {\text{MBConv}}_{{\text{n}}} ({\text{MBConv}}_{{{\text{n}} - 1}} ( \ldots ({\text{MBConv1}}({\text{X}})) \ldots )) $$

where MBConv_i_ represents the ith MBConv block in the encoder.

The decoder part of LinkNet-34 processes the high-level features extracted by the EfficientNetB7 encoder E(X). The “34” in LinkNet-34 might imply that it has 34 layers, referring to the number of convolutional layers. It performs the task of upsampling the features to the original image resolution while capturing fine-grained details for accurate segmentation. LinkNet-34 incorporates skip connections between encoder and decoder layers. The connections facilitate the model in maintaining and reusing low-level data from the encoder, so enhancing the segmentation accuracy, particularly for small and complex objects, while also retaining spatial information. The ultimate result of the LinkNet-34 model is a segmentation map that assigns each pixel to specific groups. The semantic segmentation accuracy of the model is enhanced by the rich feature representations of EfficientNetB7 and the decoder's capacity to catch intricate features. The decoder consists of upsampling the features extracted from the EfficientNetB7 encoder E(X) and subsequently applying additional convolutional layers. The result of the encoding process using the EfficientB7 encoder, denoted as E(X) and calculated according to Eq. (3), is then fed into the decoder of the LinkNet-34 model. This decoder processes the input and generates the final segmentation mask, as seen in Eq. (4):

4 $$ {\text{D}}({\text{E}}({\text{X}})) = {\text{Decoder}}({\text{E}}({\text{X}})) = {\text{Conv}}_{1} ({\text{Upsample}}({\text{Conv}}_{2} ({\text{BN}}({\text{ReLU}}({\text{E}}({\text{X}})))))) $$

Conv_1_ and Conv_2_ denote the initial and subsequent convolutional layers, Upsample denotes the operation of upsampling, and BN and ReLU denote batch normalization and activation functions, respectively.

LinkNet employs skip connections, denoted as S(X), which connect corresponding levels of the encoder and decoder.

The output of the decoder with skip connections is shown in Eq. (5):

5 $$ {\text{Final Output}} = {\text{D}}({\text{E}}({\text{X}})) + {\text{S}}(X) $$

This equation represents a basic mathematical model of the LinkNet-34 with EfficientB7 as encoder.

### Employment of different optimizers for the proposed model

In the previous section, LinkNet-34 was analysed as best semantic segmentation model with EfficientNetB7 encoder which performed best in terms of dice coefficient and jaccard index. The optimizer plays a crucial role in semantic segmentation as it is responsible for updating the model's parameters during the training process. Optimizers are algorithms that calculate what changes to make to the model's weights and biases in order to reduce the loss function, which is the distinction between the predicted and ground-truth segmentations. The choice of optimizer can significantly impact the training process and the final segmentation performance. Here, in this section, LinkNet-34 semantic segmentation model is experimented with three optimizers i.e. Adam, RMSProp and Adamax to find the best combination for their specific semantic segmentation task.

#### RMSprop optimizer analysis

**Figure 18:**
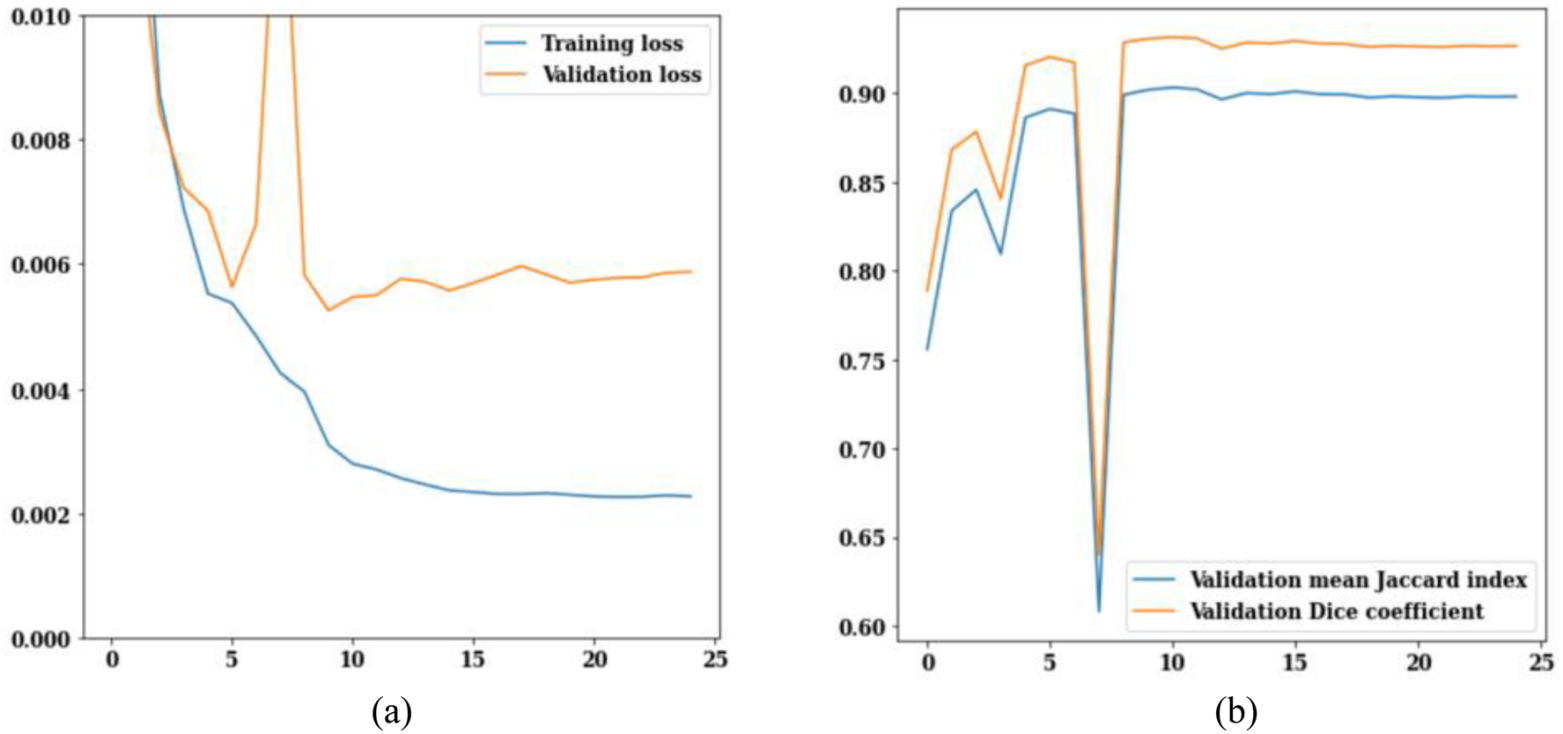
RMSProp optimizer (**a**) training and validation loss, (**b**) mean Jaccard Index and dice coefficient.

In this section, the best performing LinkNet-34 model with EfficientNetB7 as encoder is simulated with RMSprop optimizer. Values of loss, jaccard index, and dice coefficient for the RMSProp optimizer for 25 epochs are displayed in Fig. 18.

The training and validation loss values across 25 epochs are displayed in Fig. 18a. On the 25th epoch, the validation loss is 0.006 and the training loss is 0.002. The jaccard index and dice coefficient over 25 iterations are displayed in Fig. 18b. The graph value stays constant after the seventh epoch, and the jaccard index and dice coefficient reach their maximums of 0.89 and 0.91, respectively, on the 25th epoch.

#### Adamax optimizer analysis

**Figure 19:**
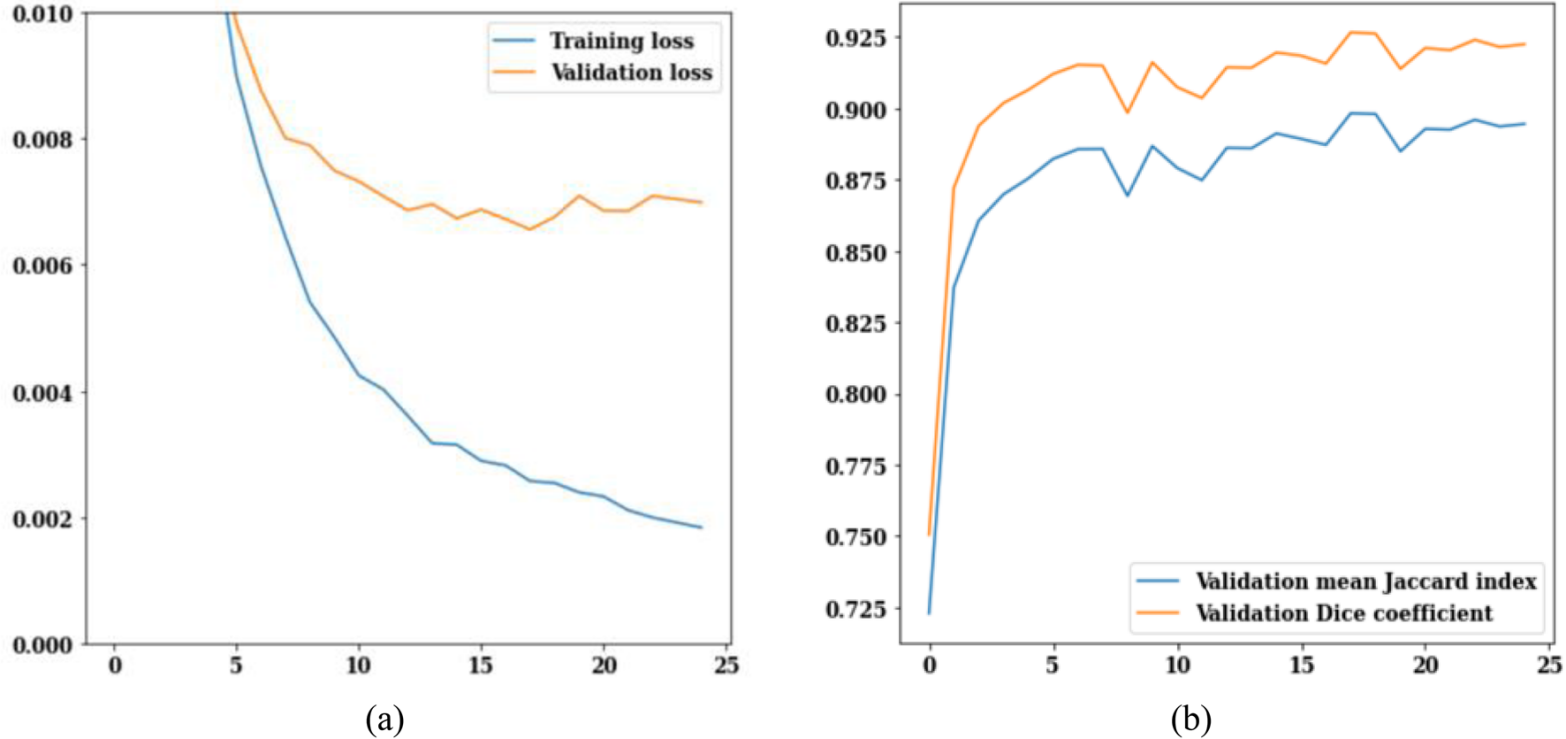
Adamax optimizer (**a**) training and validation loss, (**b**) mean Jaccard Index and dice coefficient.

In this section, the best performing LinkNet-34 model with EfficientNetB7 as encoder is simulated with Adamax optimizer. Figure 19 shows the loss, jaccard index and dice coefficient values of Adamax optimizer for 25 epochs. The values of the training and validation losses are displayed in Fig. 19a. On the 25th epoch, the validation loss is 0.007, while the training loss is 0.002 as shown in Fig. 19a. The jaccard index and dice coefficient over 25 epochs are displayed in Fig. 19b. As more epochs pass, higher values of these performance parameters are reached. On the 25th epoch, the mean jaccard index is 0.89, and the validation dice coefficient is 0.925.

#### Adam optimizer analysis

**Figure 20:**
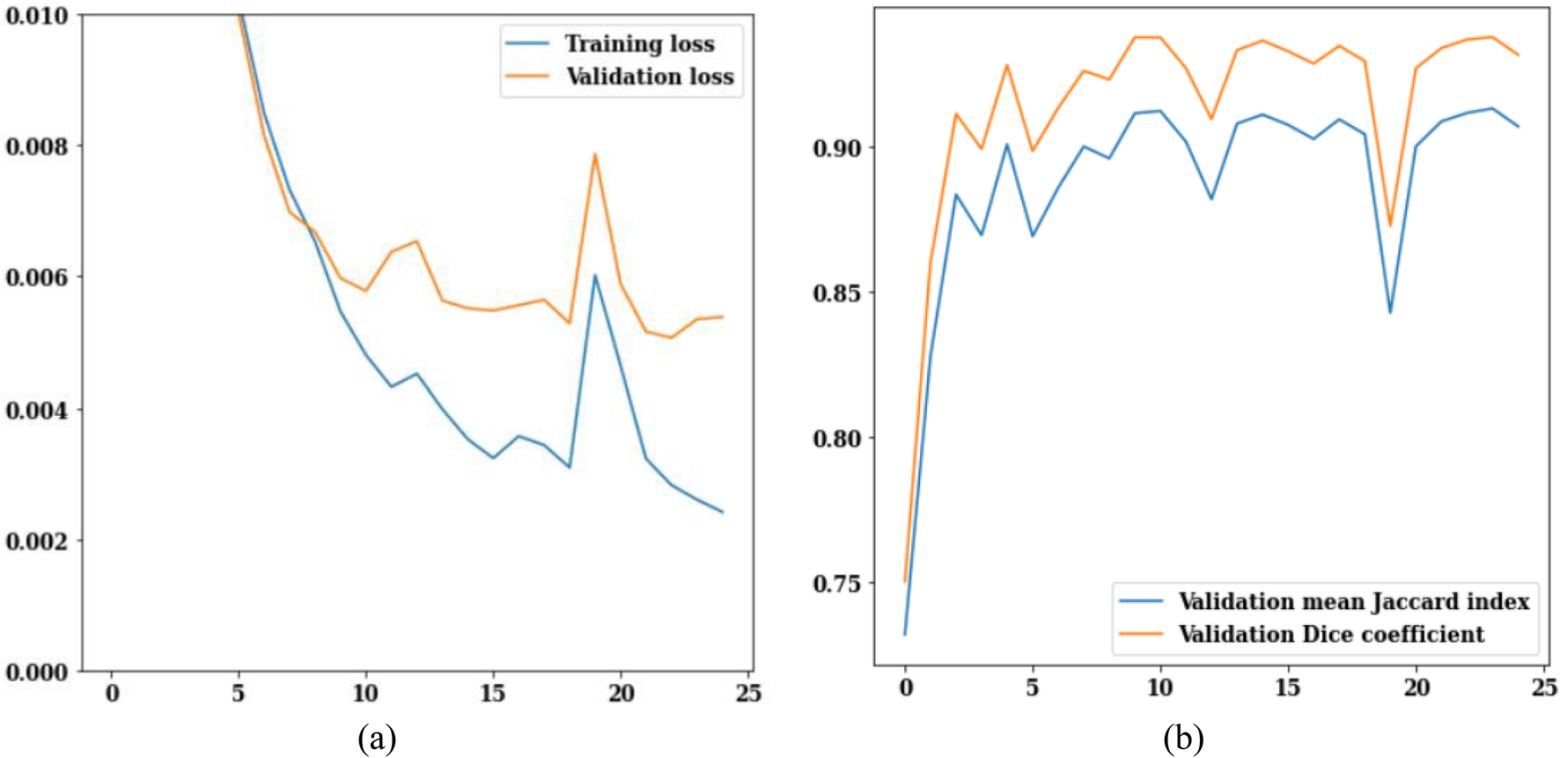
Adam optimizer (**a**) training and validation loss, (**b**) mean Jaccard Index and dice coefficient.

In this section, the best performing LinkNet-34 model with EfficientNetB7 as encoder is simulated with Adam optimizer. Figure 20 shows the loss, jaccard index and dice coefficient values of Adam optimizer for 25 epochs. The values of the training and validation losses are displayed in Fig. 20a.

On the 25th epoch, the value of training and validation loss are 0.002 and 0.006 respectively. The jaccard index and dice coefficient over 25 epochs are displayed in Fig. 20b. On the 25th epoch, the mean jaccard index is 0.90, and the validation dice coefficient is 0.91 respectively.

### Best optimizer selection

**Figure 21:**
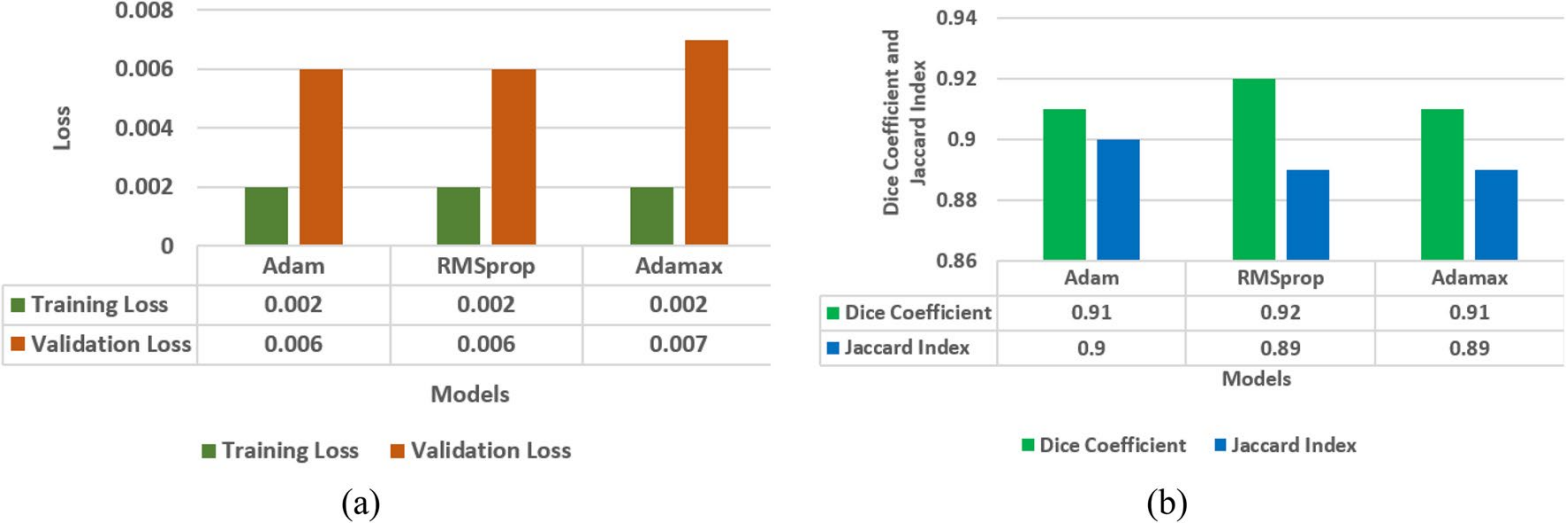
Comparison of optimizers (**a**) training and validation loss, (**b**) mean Jaccard Index and dice coefficient.

To obtain the best performing optimizer out of the three optimizers i.e. RMSProp, Adamax and Adam simulated in the previous sections, the comparison of results is performed in this section as shown in Fig. 21. It can be observed from the last sections that the values of jaccard index for three optimizers i.e. RMSProp, Adamax and Adam are 0.89, 0.89 and 0.90 and the values of dice coefficient are 0.91, 0.925 and 0.91 respectively, on the 25th epoch. It is analysed that Adamax optimizer is performing best in comparison to other optimizers having values of jaccard index and dice coefficient as 0.89 and 0.925.

This can be concluded that, LinkNet-34 model with EfficientNetB7 encoder is outperforming best with Adamax optimizer to differentiate between the predicted segmentation and the ground truth mask in semantic segmentation.

### Results on BraTs2020 dataset

**Figure 22:**
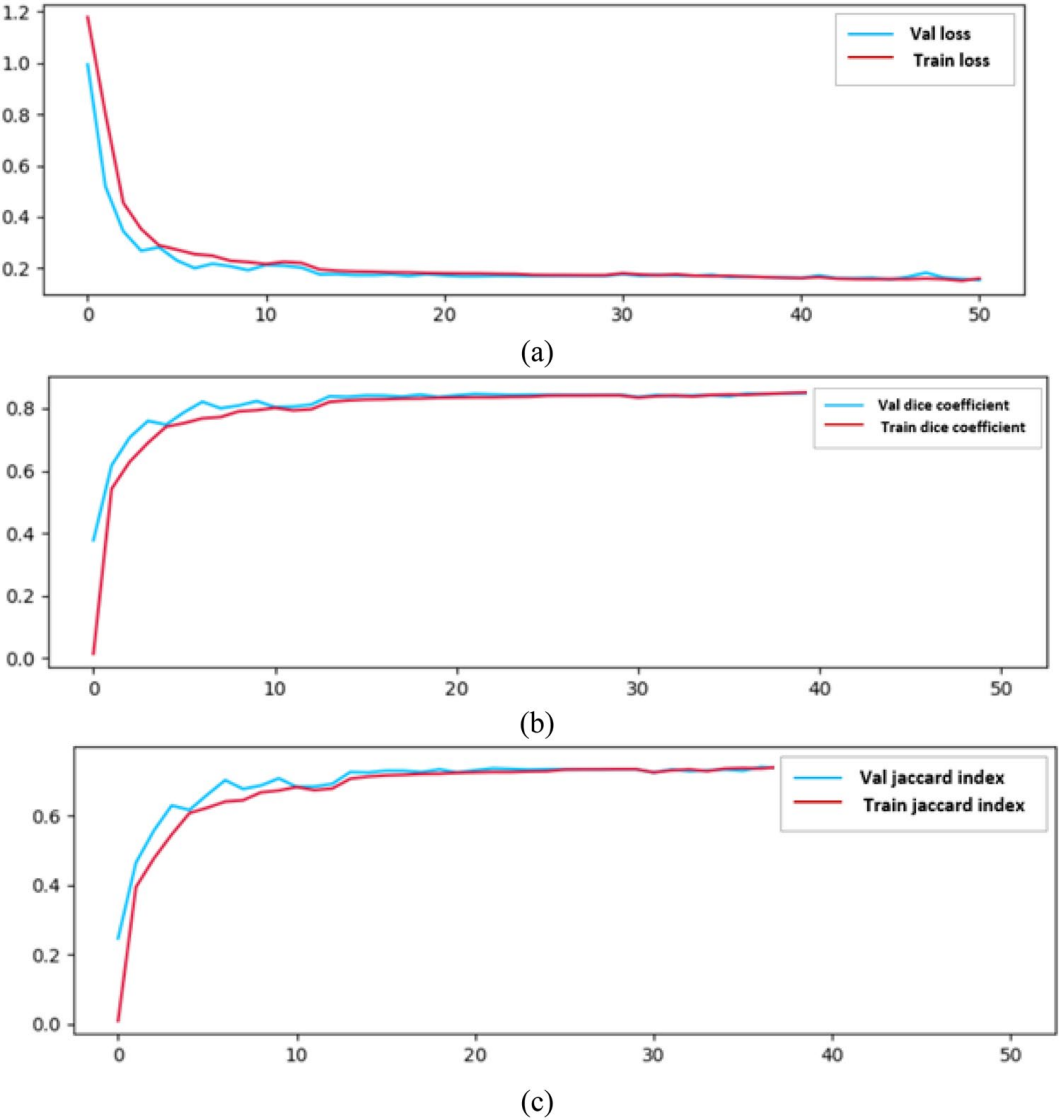
BraTs2020 dataset (**a**) loss, (**b**) dice score, (**c**) Jaccard Index.

The proposed model is implemented on BraTs2020 dataset with Adamax optimizer. The BraTs2020 dataset provides multimodal MRI images along with corresponding tumor segmentation masks. The BraTS 2020 dataset typically includes MRI volumes from different patients, each containing all four modalities (T1, T1CE, T2, and FLAIR) along with corresponding segmentation masks. The results for training loss, validation loss, training dice score, validation dice score, training jaccard score and validation jaccard score are shown in Fig. 22a–c respectively. The values of the training and validation losses are displayed in Fig. 22a. The value of training and validation loss are 0.159 and 0.153 respectively. The dice coefficient is displayed in Fig. 22b and the values of training and validation dice coefficient are 0.8526 and 0.8585 respectively. Figure 22c shows the values of training and validation jaccard index as 0.750 and 0.757.

### Visualization of segmentation results

**Figure 23:**
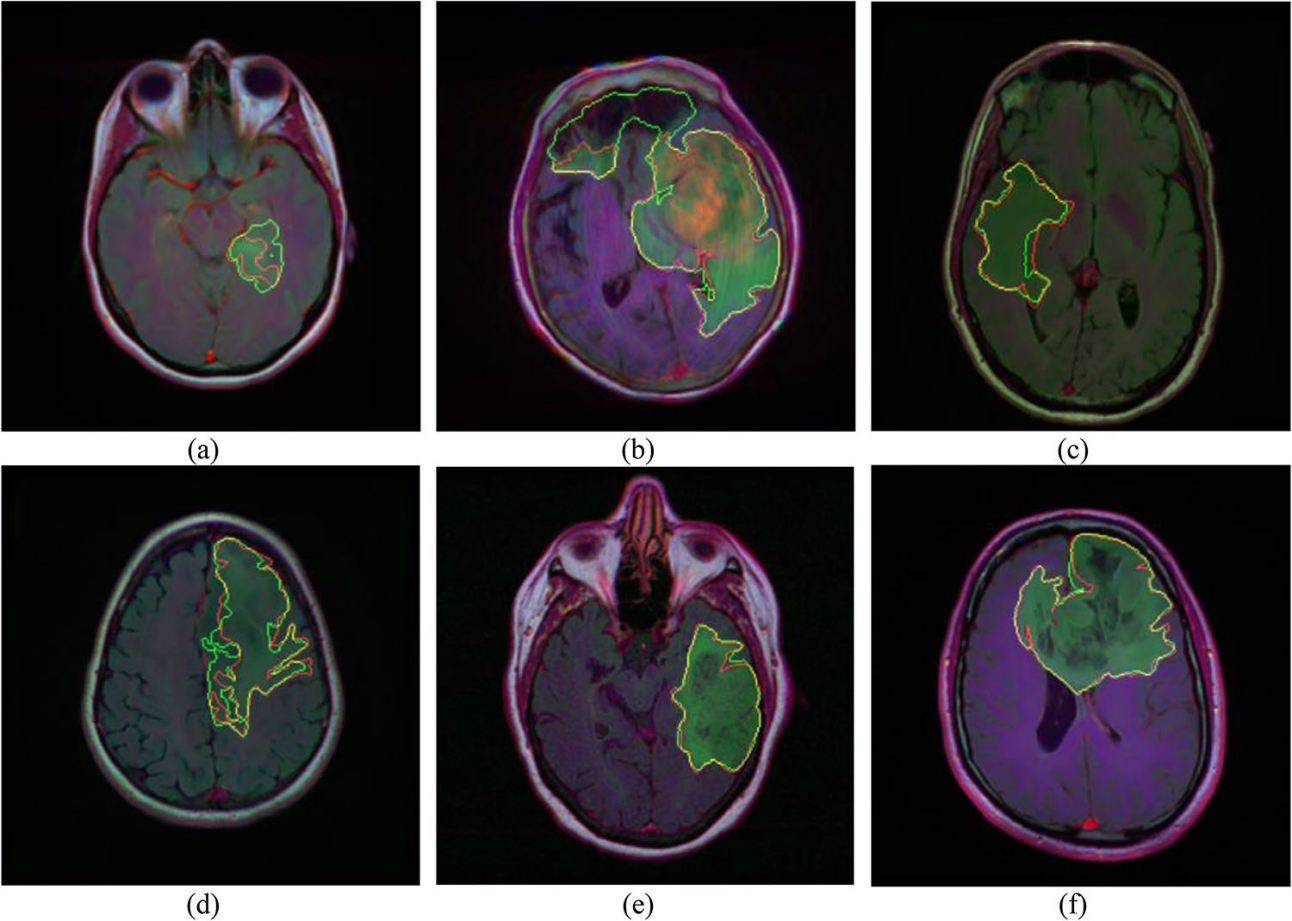
Visualization of segmented results.

In the last sections, brain tumor MRI dataset segmentation is implemented using four semantic segmentation models i.e. LinkNet-34, FPN, PSPNet, and U-Net. Out of the four models, LinkNet-34 model has outperformed best in comparison to the other three models. After that, LinkNet-34 model is simulated using four different encoders namely ResNet34, MobileNet_V2, ResNet50, and EfficientNetB7 to create a feature map that can capture high-level semantic information and spatial context from which EfficientNetB7 was concluded as best encoder in terms of dice coefficient and jaccard index. The best combination of semantic segmentation model LinkNet-34 with the best encoder i.e. EfficientNetB7 is simulated using the three different optimizers namely RMSProp, Adamax and Adam. Out of the three optimizers, Adamax optimizer is showing best results. Figure 23 shows the visualization of results of the proposed LinkNet-34 model with the combination of EfficientNetB7 encoder and Adamax optimizer. The segmented regions with red color boundary lines shows the ground truth and the green color boundary lines shows the predicted mask by the proposed model.

### Comparison with state-of-art

The state-of-art Table 1 presents various studies related to brain tumor segmentation using different techniques and datasets. The performance parameters reported for each study are mostly evaluation metrics commonly used in semantic segmentation tasks, such as Dice Coefficient, Jaccard Index, and Accuracy. From the Table 1 it can be analysed that other authors^12–20^ are working on CNN models or machine learning based models. They are not working on semantic segmentation models except Saeed et al.^39^.

**Table 1:** State-of-art comparison.

Ref./year	Dataset	Technique used	Performance parameters
^12^/2018	BRATS 2015	Cascaded CNN	Dice coefficient = 0.89
^13^/2008	Vivo brain tumors	Hybrid model	Jaccard Index = 0.69
^14^/2012	Synthetic data from Utah + in vivo data from Harvard	Cellular automata model	Dice coefficient = 0.72
^15^/2013	Web data + in vivo brain tumors	Lesion localization and segmentation model	Accuracy = 83–95%
^16^/2016	MICCAI-BRATS 2013	SVM	Dice coefficient = 0.86
^17^/2017	MICCAI-BRATS 2013	Wavelet-based features	Dice coefficient = 0.88
^18^/2015	MICCAI-BRATS 2013	Random forest model	Dice coefficient = 0.88
^19^/2015	MICCAI-BRATS 2013	Using appearance- and context-based features	Dice coefficient = 0.83
^20^/2014	MICCAI-BRATS 2013	3D CNN with 3D convolutional kernels	Dice coefficient = 0.87
^39^/2021	BraTs2020	U-Net with MobileNetV2 encoder	Dice Coefficient = 0.88
Proposed	Brain MRI, BraTs2020	LinkNet-34 semantic segmentation with EfficientNetB7 encoder	**Brain MRI** Jaccard index = 0.89, Dice coefficient = 0.92 **BraTs2020** Jaccard index = 0.75, Dice coefficient = 0.85

Saeed et al.^39^ had proposed a U-Net model with MobileNetV2 as encoder for brain tumor segmentation. Whereas, here in the proposed work, an intelligent LinkNet-34 model with EfficientNetB7 as encoder is proposed. Saeed et al. is using U-Net as decoder whereas, we are using Linknet-34 as decoder in our proposed model. U-Net have a higher computational cost due to its deeper architecture and extensive skip connections with higher number of trainable parameters whereas, LinkNet-34, designed for efficiency, aims to reduce computational cost with lower number of trainable parameters while maintaining segmentation performance. Hence, proposed model is having low computational cost due to less number of trainable parameters.

It can be concluded from the Table 1 that the proposed model for Low-Grade Gliomas (LGG) segmentation using a LinkNet-34 semantic segmentation approach with an EfficientNetB7 encoder outperforms other state-of-art models in terms of Dice Coefficient as 0.915 and a Jaccard Index as 0.89.

## Conclusions

Due to the atypical appearance and fuzziness of borders, brain tumours present a formidable challenge for segmentation. Deep learning techniques have the potential to revolutionize brain tumor segmentation. Radiologists can benefit from precise tumor segmentation by learning more about the tumor's size, location, and growth trends. These approaches are more reliable because they are less vulnerable to noise. Therefore, in this research, an encoder-based LinkNet-34 model is proposed for the semantic segmentation of brain tumors from MRI scans. The performance of the LinkNet-34 model is compared with the other three models, namely FPN, U-Net, and PSPNet. Further, the performance of EfficientNetB7 that is used as encoder in LinkNet-34 model has been compared with three encoders namely ResNet34, MobileNet_V2, and ResNet50. After that, the proposed model is optimized using three different optimizers such as RMSProp, Adamax and Adam. The Adamax optimizer has outperformed in comparison to other two optimizers with the values of the validation mean jaccard index and the validation dice coefficient as 0.89 and 0.915 respectively.

Therefore, encoder based LinkNet-34 model can give radiologists more precise details regarding the tumor's location and growth tendencies. With this data in hand, doctors can determine the most effective therapy options for the patient.

## Data Availability

The data is publicly accessible as “Brain MRI Segmentation” and the link is: https://www.kaggle.com/datasets/mateuszbuda/lgg-mri-segmentation.
